# Synergistic Effects Between Chitooligosaccharide–Epigallocatechin Gallate Conjugate and Nisin on Inhibition of Seafood-Associated *Vibrio* spp., *Aeromonas* spp., and *Shewanella* spp.

**DOI:** 10.3390/foods15173072

**Published:** 2026-08-30

**Authors:** Suriya Palamae, Pitima Sinlapapanya, Jirayu Buatong, Natchaphol Buamard, Yu Fu, Bin Zhang, Hui Hong, Soottawat Benjakul

**Affiliations:** 1 International Center of Excellence in Seafood Science and Innovation (ICE-SSI), Faculty of Agro-Industry, Prince of Songkla University, Hat Yai 90110, Songkhla, Thailand; suriya.pal@psu.ac.th (S.P.); pitimasinlapapanya@gmail.com (P.S.); jirayu.b@psu.ac.th (J.B.); natchaphol.b@psu.ac.th (N.B.); 2 College of Food Science, Southwest University, No. 2. Tiansheng Road, Beibei District, Chongqing 400715, China; fuy987@swu.edu.cn; 3 Key Laboratory of Health Risk Factors for Seafood of Zhejiang Province, College of Food Science and Pharmacy, Zhejiang Ocean University, Zhoushan 316022, China; zhangbin@zjou.edu.cn; 4 Beijing Laboratory for Food Quality and Safety, College of Food Science and Nutritional Engineering, China Agricultural University, Beijing 100083, China; hhong@cau.edu.cn; 5 Department of Food and Nutrition, Kyung Hee University, 26 Kyungheedae-ro, Dongdaemun-gu, Seoul 02447, Republic of Korea

**Keywords:** Asian moon scallop, food safety, foodborne pathogen, food spoilage, ready-to-cook products

## Abstract

Ready-to-cook Asian moon scallop (RAM) may harbor seafood-associated spoilage and opportunistic pathogenic bacteria, leading to quality deterioration and food safety concerns. This study investigated the microbiological diversity, virulence-associated phenotypes, and antibacterial activity of chitooligosaccharide–epigallocatechin gallate conjugate (CEGC) combined with nisin against seafood-associated bacteria isolated from RAM. A total of 102 bacterial isolates were subjected to identification by MALDI-TOF/MS, with *Vibrio*, *Aeromonas*, and *Shewanella* as the predominant genera. Several isolates exhibited proteolytic and hemolytic activities, indicating spoilage potential and virulence-associated traits. Multivariate analysis showed that bacterial isolates differed widely in their antimicrobial susceptibility and virulence-associated phenotypes. Checkerboard assays demonstrated synergistic effects between CEGC and nisin against *Vibrio parahaemolyticus* and *Vibrio fluvialis* (FICI = 0.50). The additive effects were observed for the remaining strains, except an indifferent effect (FICI = 1.06) was found for *A. hydrophila*. The combined treatment markedly inhibited bacterial growth and biofilm formation, achieving approximately 95% biofilm reduction in *V. parahaemolyticus*. Confocal laser scanning microscopy and scanning electron microscopy revealed severe membrane disruption, increased permeability, cellular deformation, and collapse of biofilm architecture. Furthermore, CEGC–nisin effectively suppressed *V. parahaemolyticus* growth in cold-stored RAM. These findings demonstrate that CEGC combined with nisin is a promising natural antimicrobial strategy for improving seafood safety and reducing microbial spoilage in ready-to-cook seafood products.

## 1. Introduction

Asian moon scallop or Asian moon shell (*Amusium pleuronectes*) is an economically important marine bivalve widely distributed throughout Indo-Pacific coastal regions, including Thailand, China, Japan, Indonesia, the Philippines, and Australia [1]. Owing to its delicate flavor, tender texture, and high nutritional value, this bivalve has become one of the most commercially valuable seafood in Southeast Asia. In Thailand, *A. pleuronectes* is extensively harvested and marketed as a ready-to-cook seafood product. However, similar to other bivalve mollusks, Asian moon scallop is highly susceptible to microbial contamination. Because of its filter-feeding behavior, the accumulation of pathogenic and spoilage microorganisms from surrounding aquatic environments generally occurs [2]. Consumption of raw or under-cooked contaminated shellfish has frequently been associated with foodborne illnesses and seafood spoilage, thereby raising significant concerns regarding seafood safety and quality maintenance.

Among the major bacterial groups associated with seafood contamination, *Vibrio*, *Aeromonas*, and *Shewanella* species are commonly detected in marine and estuarine environments [3]. *Vibrio* species, particularly *V. parahaemolyticus*, *V. vulnificus*, and *V. cholerae*, are recognized as important seafood-borne pathogens responsible for gastroenteritis, septicemia, and wound infections in humans [4]. Other emerging pathogenic species such as *V. alginolyticus*, *V. mimicus*, *V. harveyi*, and *V. fluvialis* have also been increasingly reported in seafood products [5]. The Centers for Disease Control and Prevention estimated that vibriosis accounts for approximately 80,000 illnesses annually in the United States, in which seafood consumption was identified as the major transmission route [6]. In addition, *Aeromonas* species, including *A. hydrophila*, *A. veronii*, and *A. caviae*, are widely distributed in aquatic ecosystems and are capable of growing under refrigerated conditions [7]. These bacteria are associated with gastroenteritis, bacteremia, septicemia, skin infections, and other opportunistic infections, especially in immunocompromised individuals [8,9,10]. *Shewanella* species are generally recognized as predominant spoilage bacteria in seafood. These bacteria are able to produce undesirable compounds such as ammonia, hydrogen sulfide, and sulfur-containing volatile compounds, contributing to off-odor and quality deterioration during storage [11].

The pathogenicity and persistence of these microorganisms are strongly related with various virulence factors, including hemolysins, proteases, adhesins, enterotoxins, lipases, aerolysins, and biofilm-forming ability [12]. Basically, biofilm formation enhances bacterial survival under adverse environmental conditions and increases resistance to conventional antimicrobial treatments. Moreover, the emergence of antibiotic-resistant *Vibrio* and *Aeromonas* species has become a major public health concern worldwide. Several studies have reported the occurrence of multidrug-resistant *Vibrio* spp. in seafood products from different countries, emphasizing the urgent need for effective natural antimicrobial strategies to control seafood-associated pathogens and spoilage microorganisms.

To maintain seafood quality and safety, refrigeration and hygienic handling are commonly implemented during processing and storage. Nevertheless, psychrotrophic spoilage bacteria can still proliferate under chilled conditions, resulting in quality deterioration and shortened shelf life. In recent years, an increasing consumer demand for minimally processed and preservative-free seafood products has encouraged the development of natural antimicrobial agents as alternatives to synthetic preservatives. Among these, chitooligosaccharides (COS), derived from chitosan hydrolysis, have attracted considerable attention because of their biocompatibility, biodegradability, and broad-spectrum biological activities, including antioxidant and antimicrobial properties [13]. Conjugation of COS with phenolic compounds has been demonstrated to further enhance their antimicrobial activity [14]. Epigallocatechin gallate (EGCG), a major catechin derivative found in green tea, possesses potent antioxidant and antimicrobial activities. When COS was conjugated with EGCG, antimicrobial efficacy of resulting conjugate (CEGC) against several foodborne pathogens and spoilage bacteria was improved [14]. Additionally, nisin, a natural bacteriocin produced by *Lactococcus lactis*, has been widely used in food preservation due to its strong antibacterial activity and safety status [15]. Although nisin is generally more effective against Gram-positive bacteria, its antimicrobial performance against Gram-negative bacteria can be enhanced when combined with membrane-disrupting agents or synergistic compounds. Charest et al. [16] reported inhibitory activity of nisin against a broad range of Gram-negative bacteria and suggested that synergistic supplements can enhance nisin sensitivity by facilitating access to bacterial membranes. Previous studies also reported that combinations of nisin with natural antimicrobial compounds, such as tea polyphenols and rhamnolipids, showed synergistic antibacterial effects against *Bacillus cereus* in pasteurized whole milk and cooked rice through membrane disruption, intracellular leakage, and reactive oxygen species accumulation [17]. Therefore, combining CEGC with nisin might provide a promising hurdle approach for controlling seafood-associated microorganisms, causing illness and quality loss.

Despite the increasing interest in natural antimicrobial systems, CEGC and nisin have been investigated individually for their antibacterial activities against food-associated microorganisms [13,14,16]. However, studies investigating their synergistic inhibitory effects against seafood-associated *Vibrio*, *Aeromonas*, and *Shewanella* species remain limited [18], particularly in ready-to-cook Asian moon scallop (RAM) products. Therefore, this study aimed to investigate the synergistic antibacterial activity between CEGC and nisin against pathogenic and spoilage bacteria isolated from Asian moon scallop. The findings of this study could provide valuable insights into the development of natural antimicrobial strategies for reducing the risk of foodborne illnesses associated with bivalve consumption as well as prolonging shelf life of perishable bivalves.

## 2. Materials and Methods

### 2.1. Chemicals, Reagents, Chitooligosaccharide–Epigallocatechin Gallate Conjugate (CEGC) and Microbial Media

Unless otherwise indicated, all analytical-grade chemicals and reagents were obtained from Sigma-Aldrich (St. Louis, MO, USA), while microbiological media were purchased from Oxoid™ (Thermo Fisher Scientific, Waltham, MA, USA). Shrimp shell-derived chitosan (molecular weight of 2100 kDa; degree of deacetylation of 85%), characterized by gel permeation chromatography and 1H-NMR spectroscopy, respectively, was supplied by Marine Bio Resources Co., Ltd. (Samut Sakhon, Thailand). Epigallocatechin gallate (EGCG; ≥99% purity) was procured from Xi’an Julong Bio-Tech Co., Ltd. (Xi’an, China). The chitooligosaccharide–epigallocatechin gallate conjugate (CEGC) was synthesized using a free radical grafting technique based on the method described by Mittal et al. [13]. Nisin from *Lactococcus lactis* (≥900 IU/mg; CAS No. 1414-45-5) was acquired from Sigma-Aldrich (St. Louis, MO, USA) and used at concentrations of 196−10,000 µg/mL, expressed as the mass concentration of the commercial nisin preparation. It was dissolved in 0.01 M HCl and subsequently diluted with sterile distilled water to the desired concentrations before use. All prepared nisin solutions were stored at 4 °C and used within 24 h.

### 2.2. Sample Collection and Preparation

A total of 25 ready-to-cook chilled Asian moon scallop (RAM, *Amusium pleuronectes*) samples were obtained from local retail markets in Songkhla Province, Thailand. Some samples were bought from online distributors located in several coastal regions of Thailand along both the Gulf of Thailand and the Andaman Sea; all samples were wild-caught. Samples purchased through online platforms were delivered under well-controlled refrigerated conditions (4 °C) at with approximately 12–15 h of transportation to a local supplier in Hat Yai. The average sample weight was 20.49 ± 2.90 g. Immediately after purchase or receipt, all samples were packed in polyethylene bag, placed in insulated polystyrene boxes containing crushed ice and transported to the laboratory for subsequent analyses.

### 2.3. Microbiological Quality Assessment and Bacterial Isolation

Microbiological quality of RAM samples was evaluated following the procedures outlined by the U.S. Food and Drug Administration Bacteriological Analytical Manual (BAM) [19], with slight modifications as described by Palamae et al. [20]. The edible portions (EPs) from approximately five individual scallops were aseptically excised and pooled to obtain approximately 25 g of EP. Using sterile forceps and sterile scissors, the EPs were aseptically cut into small pieces and thoroughly mixed to obtain a uniform sample while minimizing the risk of cross-contamination. The average EP weight was 5.13 ± 0.64 g per scallop. Subsequently, 25 g of the pooled EP was homogenized with 225 mL of sterile 0.85% saline solution using a homogenizer at 300 rpm for 2 min (Stomacher 400 Seaward Medicals, Worthing, UK). The resulting homogenate was serially diluted in sterile 0.85% saline solution prior to microbiological enumeration. For aerobic plate count (APC), an aliquot of appropriate dilutions (100 µL) was spread onto plate count agar (CM0463, Oxoid™, Thermo Fisher Scientific, Waltham, MA, USA). Presumptive *Aeromonas* spp. were enumerated on *Aeromonas* medium base agar (CM0833, Oxoid™) supplemented with ampicillin selective supplement (SR0136, Oxoid™). For isolation, presumptive *Aeromonas* colonies exhibiting typical morphology, characterized by green opaque colonies with dark centers and diameters ranging from 1–2 mm, were selected from the selective medium for further analysis.

Enumeration of *V. parahaemolyticus*, other *Vibrio* spp., and presumptive *Shewanella* spp. was performed using thiosulfate citrate bile salts sucrose (TCBS) agar (CM0333, Oxoid™). On TCBS agar, *V. parahaemolyticus* appeared as blue-green colonies, whereas sucrose-fermenting *Vibrio* spp. produced yellow colonies. Presumptive *Shewanella* spp. were characterized by the formation of small black colonies, resulting from hydrogen sulfide production and iron sulfide precipitation. All inoculated plates were incubated at 35 ± 2 °C for 24–48 h, except for *Aeromonas* spp., which were incubated at 30 ± 2 °C for 24 h. Microbial load was expressed as log CFU/g sample.

### 2.4. Bacterial Identification

Representative presumptive bacterial isolates obtained from selective media were further purified prior to identification. A total of 64 presumptive *Vibrio* isolates were selected and subsequently purified on tryptic soy agar (TSA; CM0131B, Oxoid™) supplemented with 3% NaCl (*w*/*v*) (TSA-N) to promote the growth of halophilic bacteria. In addition, 18 presumptive *Shewanella* and 20 presumptive *Aeromonas* isolates were selected based on their characteristic colony morphology on TCBS agar and *Aeromonas* medium base agar supplemented with ampicillin selective supplement, respectively. Both presumptive *Shewanella* and *Aeromonas* isolates were purified on regular TSA.

Species identification was performed using Matrix-Assisted Laser Desorption/Ionization Time-of-Flight Mass Spectrometry (MALDI-TOF/MS) with a MALDI Biotyper system (microflex LT; Bruker Daltonik GmbH, Bremen, Germany) [21]. Spectral profiles were acquired in positive linear mode over a mass range of 2–20 kDa using FlexControl software (version 3.4) and subsequently analyzed using Biotyper Realtime Classification software (version 3.1). Identification scores of ≥2.0 were considered reliable for species-level identification, whereas the scores between 1.8 and 1.99 represented genus-level identification. However, the scores of <1.8 were regarded as unreliable [20]. Final species identified was based on the highest confidence score obtained for each isolate.

### 2.5. Determination of Minimum Inhibitory Concentration (MIC) and Minimum Bactericidal Concentration (MBC) of CEGC and Nisin

The minimum inhibitory concentration (MIC) and minimum bactericidal concentration (MBC) of CEGC and nisin against various selected bacteria comprising six reference strains, including *V. parahaemolyticus* ATCC 17802, *V. fluvialis* BL186, *V. alginolyticus* DMST 14800, *Aeromonas hydrophila* ATCC 7966, *A. veronii* ATCC 35624, and *Shewanella putrefaciens* JCM 20190, together with 102 bacterial isolates recovered from RAM samples, were determined using the broth microdilution method [22,23]. Briefly, bacterial suspensions were prepared in sterile 0.85% NaCl solution and adjusted to 0.5 McFarland standard (approximately 10^8^ CFU/mL). The suspensions were subsequently diluted 200-fold in Mueller–Hinton broth (MHB; Difco™, Baltimore, MD, USA) with or without 3% NaCl supplementation to obtain a final concentration of approximately 10^6^ CFU/mL.

For the MIC assay, CEGC and nisin were prepared in MHB at 2× working concentrations, ranging from 196 to 10,000 µg/mL. An aliquot (100 µL) of each antimicrobial agent with 2× working concentration was mixed with 100 µL of the standardized bacterial suspension (approximately 10^6^ CFU/mL) in sterile 96-well microtiter plates, giving a final volume of 200 µL per well. Consequently, the final concentrations of CEGC and nisin in the wells ranged from 98 to 5000 µg/mL, while the final bacterial inoculum concentration was approximately 5 × 10^5^ CFU/mL per well. The inoculated microplates were incubated at 37 °C for 24 h under static conditions. MIC value was defined as the lowest concentration of the tested compounds, showing no visible bacterial growth after incubation. All determinations were conducted in triplicate.

For MBC determination, 100 µL aliquots from wells showing no visible growth were directly spread onto Mueller–Hinton agar plates and incubated at 37 °C for 24 h. The MBC was defined as the lowest concentration showing no detectable bacterial colonies after direct plating. The potential influence of antimicrobial carryover associated with the direct-plating approach was also considered when interpreting the MBC results.

### 2.6. Characterization of Virulence-Associated Phenotypes

A total of 102 representative isolates of *Vibrio* spp., *Aeromonas* spp., and *Shewanella* spp. isolated from RAM samples, together with six reference strains, *V. parahaemolyticus* ATCC 17802, *V. fluvialis* BL186, *V. alginolyticus* DMST 14800, *A. hydrophila* ATCC 7966, *A. veronii* ATCC 35624, and *S. putrefaciens* JCM 20190 were evaluated for virulence-associated phenotypic traits.

#### 2.6.1. Proteolytic Activity

Protease production was determined using skim milk agar as a substrate for casein hydrolysis. For *Aeromonas* spp. and *Shewanella* spp., the medium consisting of 1.5% (*w*/*v*) agar supplemented with 1% (*w*/*v*) skim milk was used. For *Vibrio* isolates, the medium was additionally supplemented with 3% (*w*/*v*) NaCl. Bacterial suspensions were prepared in sterile 0.85% NaCl solution and adjusted to a turbidity equivalent to 0.5 McFarland standard as described previously. Subsequently, 2-µL of aliquot were spotted onto the agar surface and incubated at 37 °C for 24 h. Proteolytic activity was assessed in triplicate. The formation of clear hydrolysis zones surrounding bacterial colonies indicated the extracellular protease-mediated degradation of casein.

#### 2.6.2. Hemolytic Activity

Hemolytic activity of *Aeromonas* spp. and *Shewanella* spp. isolates was evaluated on blood agar following the procedure described by Santajit et al. [24]. Briefly, 2 µL of bacterial suspension adjusted to 0.5 McFarland standard was inoculated onto blood agar plates and incubated at 37 °C for 24 h. Hemolytic activity was identified by the occurrence of transparent zones surrounding colonies.

#### 2.6.3. Kanagawa Phenomenon Test

The Kanagawa phenomenon assay for *Vibrio* isolates was conducted according to the method described by Zhang et al. [25]. Briefly, 2 µL of bacterial suspension adjusted to 0.5 McFarland standard was inoculated onto Wagatsuma agar supplemented with 5% rabbit red blood cells. The inoculated plates were incubated statically at 37 °C for 24 h. A positive KP reaction was reflected by the appearance of distinct β-hemolytic zones surrounding the inoculation site. The diameter of the hemolytic zone was subsequently recorded.

### 2.7. Checkerboard Synergy Assay

The synergistic antibacterial activity between CEGC and nisin against six reference strains was assessed using the checkerboard broth microdilution method in 96-well microtiter plates. Both antimicrobial agents were subjected to serial dilutions ranging from 4 × MIC to 1/32 × MIC in MHB with or without 3% NaCl supplementation. Bacterial suspensions were adjusted to approximately ~10^6^ CFU/mL and inoculated into wells containing different combinations of CEGC and nisin. After incubation at 37 °C for 24 h, antibacterial interactions were assessed by calculating the fractional inhibitory concentration index (FICI) using the following equation:

$$\mathrm{FICI}\;=\;\left(\frac{{\mathrm{MIC}}_{A/B}}{{\mathrm{MIC}}_{A}}\right)\;+\;\left(\frac{{\mathrm{MIC}}_{B/A}}{{\mathrm{MIC}}_{B}}\right)$$

where MIC_A_ and MIC_B_ are the MIC values of compounds A and B used alone, while MIC_(A/B)_ and MIC_(B/A)_ represent the MIC values of combined compounds. FICI values of ≤0.5, >0.5–1.0, >1.0–2.0, and >2.0 represented synergistic, additive, indifferent and antagonistic interaction, respectively. All assays were performed in triplicate.

### 2.8. Mode of Action

#### 2.8.1. Inactivation of Seafood-Associated Bacteria by CEGC and Nisin

The antibacterial efficacy of combined CEGC and nisin against seafood-associated bacteria including six reference strains was evaluated using an inactivation assay following the method described by Wang et al. [26]. Overnight bacterial cultures were diluted to approximately ~10^6^ CFU/mL in tryptic soy broth (TSB) supplemented with 3% NaCl (TSB-N) for *Vibrio* strains or TSB for *Aeromonas* and *Shewanella* strains. Subsequently, CEGC and nisin were added to bacterial suspensions at their respective strain-specific combined MIC concentrations determined by the checkerboard assay (Table 1). After gentle mixing, the inoculated tubes were incubated at 37 °C under continuous shaking conditions (200 rpm) (Model G-25R, New Brunswick Scientific Co., Edison, NJ, USA). Viable bacterial populations were enumerated at 0 and 24 h using the spread plate technique, and the antibacterial activity was expressed as changes in viable cell counts (log CFU/mL) after treatment.

#### 2.8.2. Biofilm Crystal Violet (CV) Staining

Biofilm formation inhibition was assessed using the crystal violet (CV) staining assay following the method of Wang et al. [27], with slight modifications. Overnight six reference strain cultures were adjusted to approximately 10^6^ CFU/mL in TSB-N and TSB. Aliquots were transferred into sterile 96-well microplates and incubated statically at 37 °C for 24 h to allow biofilm development.

Following incubation, planktonic cells were removed, and adherent biofilms were gently washed three times with sterile phosphate-buffered saline (PBS). The attached biofilms were stained with 0.1% crystal violet for 15 min, rinsed with sterile distilled water, and air-dried. The retained dye was solubilized with absolute ethanol, and biofilm biomass was quantified by measuring the absorbance at 570 nm (A_570_) using a FLUOstar Omega microplate reader (BMG Labtech GmbH, Ortenberg, Germany). Bacterial growth was additionally evaluated by measuring the absorbance of planktonic cultures at 600 nm (A_600_) using the same instrument.

#### 2.8.3. Cell Membrane Permeability

The effects of CEGC, nisin and their combination on bacterial cell membrane permeability were evaluated using propidium iodide (PI) staining coupled with confocal laser scanning microscopy (CLSM) [28]. Bacterial suspensions (~10^6^ CFU/mL) of six reference strains were treated with CEGC and nisin alone or their combination under the designated conditions, while untreated cells served as the control. Following the treatments, 10 µL of bacterial suspension was mixed with 990 µL of sterile saline solution containing 3 µL of 1.0 mg/mL propidium iodide (PI) solution (Invitrogen, Thermo Fisher Scientific, Carlsbad, CA, USA). The mixtures were incubated in the dark at room temperature for 20 min. The stained samples were subsequently observed using a confocal laser scanning microscope (TI-EAI, Nikon, Tokyo, Japan). Cells exhibiting red fluorescence were considered membrane-compromised. Basically, PI penetrates only cells with damaged cytoplasmic membranes and binds to intracellular nucleic acids. The fluorescence intensity and distribution were used to evaluate membrane permeability induced by tested compounds.

#### 2.8.4. Scanning Electron Microscopic (SEM) Analysis

Morphological alterations of six reference strains treated with CEGC and nisin, either individually or in combination, were examined using scanning electron microscopy (SEM). Bacterial cultures in the logarithmic growth phase (approximately 10^6^ CFU/mL) were exposed to the selected treatments at 37 °C for 18 h, while untreated cells served as the control. After treatment, bacterial cells were harvested by centrifugation (4500× *g*, 10 min) (Eppendorf 5430R centrifuge, Hamburg, Germany), washed three times with 0.1 M phosphate-buffered saline (PBS), and fixed with 2.5% (*w*/*v*) glutaraldehyde overnight at 4 °C. The fixed cells were subsequently dehydrated through a graded ethanol series (50%, 60%, 70%, 80%, 90%, and 100% ethanol), in which each concentration was applied for 10 min. The samples were then subjected to two changes of absolute ethanol to ensure complete dehydration and subsequently dried using a critical point dryer with CO_2_ as the transitional fluid. The dried samples were mounted onto aluminum stubs using conductive carbon tape, sputter-coated with gold, and examined using a scanning electron microscope (FEI Quanta 400-ESEM FEG, Hillsboro, OR, USA).

#### 2.8.5. Confocal Laser Scanning Microscopic (CLSM) Analysis of Biofilm Formation

Among the tested isolates, *V. parahaemolyticus* ATCC 17802, which exhibited the highest biofilm formation, was selected for CLSM analysis. The inhibitory effects of CEGC, nisin and their combination on biofilm formation were evaluated according to Palamae et al. [29]. Bacterial suspensions (~10^6^ CFU/mL) of *V. parahaemolyticus* ATCC 17802 were treated with CEGC and nisin alone or their combination under the designated conditions in 4-wells cell culture chamber slides (SPL Life Sciences Co., Ltd., Gyeonggi-do, Korea). Untreated cultures grown in TSB-N served as the control. The cultures were incubated statically at 37 °C for 48 h to allow biofilm formation. Following incubation, planktonic cells were removed, and adherent biofilms were gently washed twice with sterile 0.1 M phosphate-buffered saline (PBS; pH 7.2). The biofilms were then stained with SYTO 9 fluorescent dye (Invitrogen, Thermo Fisher Scientific, Waltham, MA, USA) in the dark for 30 min and rinsed with sterile PBS to remove the excess stain. SYTO 9 was selected to visualize the spatial architecture, distribution, and thickness of biofilms by fluorescence microscopy, whereas crystal violet staining is primarily used for the quantitative determination of total biofilm biomass. Biofilm structures were observed using a Zeiss LSM 800 Airyscan confocal laser scanning microscope equipped with ZEN 2.5 software (Carl Zeiss, Jena, Germany). Fluorescence images were obtained at an excitation wavelength of 488 nm and emission range of 500–550 nm using a 100× objective lens. Two-dimensional and three-dimensional biofilm architectures, including biofilm distribution and thickness, were analyzed from z-stack images. All experiments were performed in triplicate.

### 2.9. Inhibition of V. parahaemolyticus in Asian Moon Scallop

*V. parahaemolyticus* ATCC 17802 suspension (100 µL, 10^7^ CFU/mL) was added into ~10 g of RAM edible portion to attain the final concentration of 10^5^ CFU/g. RAM samples were mixed well under sterile conditions and left for 15 min. Subsequently, CEGC, nisin and their combination were added and mixed thoroughly. The final concentrations of CEGC and nisin in the RAM samples were 313 µg/mL and 2500 µg/mL, respectively, when applied individually. For the combined treatment of RAM samples, the final concentrations were 78 µg/mL CEGC and 625 µg/mL nisin, respectively. The sample added with sterile water was named as the control. RAM samples without bacterial suspension or any antimicrobial agent were used and considered as ‘native control’. The treated samples and the controls (10 g each) were placed in the polyethylene bag and seal before being kept at 4 °C for 3 days. The RAM samples (10 g) were added with 0.85% NaCl (*w*/*v*) solution (90 mL) and homogenized (230 rpm for 30 s) using a stomacher (Stomacher 400 Seaward Medicals, Worthing, UK). The number of live *V. parahaemolyticus* was counted for bacterial colonies growing on TCBS agar plates. The count at Day 0 likely represented the recoverable *V. parahaemolyticus* after addition of antimicrobial agents, not the original inoculum level in the RAM samples. Some reduction in *V. parahaemolyticus* might occur immediately after exposure to the antimicrobial treatment.

### 2.10. Multivariate Relationships

Principal component analysis (PCA) biplots and Pearson correlation heatmap analysis were performed to investigate the relationships among antimicrobial susceptibility (MIC and MBC of CEGC and nisin) and virulence-associated phenotypes (hemolytic and proteolytic activities) of the 102 seafood-associated bacterial isolates. Multivariate statistical analyses were conducted using R software (version 4.3.1; R Foundation for Statistical Computing, Vienna, Austria).

### 2.11. Experimental Design and Statistical Analyses

A completely randomized design (CRD) was used for all experiments. The dependent variables and experimental factors were defined according to the objectives of each experiment. Data were analyzed using one-way or two-way analysis of variance (ANOVA), as appropriate, followed by Tukey’s multiple-comparison test at a significance level of *p* < 0.05. All experiments were performed using three independent biological replicates (*n* = 3). For technical replicates, their values obtained were averaged before statistical analysis, while the biological replicate was considered the experimental unit. Results are presented as mean ± standard deviation (SD).

## 3. Results and Discussion

### 3.1. Bacterial Diversity and Antimicrobial Susceptibility of Seafood-Associated Spoilage and Pathogenic Bacteria

**Figure 1:**
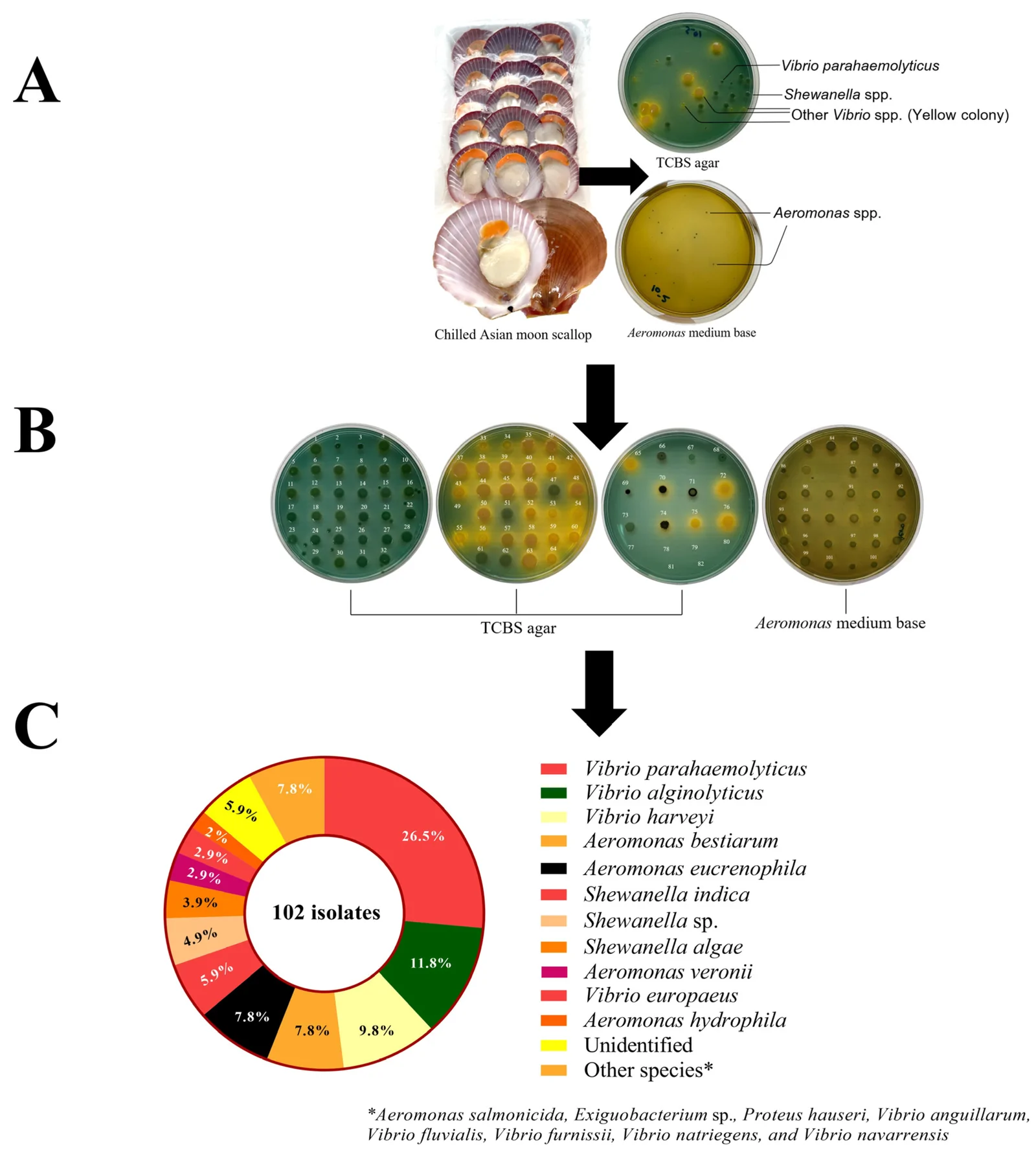
Composition and identification of selected seafood-associated bacterial isolates. (**A**) Proportions of presumptive bacterial groups isolated using selective culture media. (**B**) Representative colony morphologies of 102 isolates of *Vibrio* spp., *Shewanella* spp., and *Aeromonas* spp. grown on thiosulfate–citrate–bile salts–sucrose (TCBS) agar and *Aeromonas* medium base supplemented with ampicillin. (**C**) Bacterial composition and species identification of the isolates determined by matrix-assisted laser desorption/ionization time-of-flight mass spectrometry (MALDI-TOF MS). Data are presented as percentages of the selected isolates obtained from seafood samples.

The average aerobic plate count (APC) of RAM samples was 5.7 ± 0.4 log CFU/g, indicating a relatively high microbial load of raw material. Among the investigated bacterial groups, presumptive *Shewanella* spp. showed the highest population (5.9 ± 0.5 log CFU/g), followed by *Aeromonas* spp. (5.6 ± 0.3 log CFU/g). *Vibrio parahaemolyticus* and sucrose-fermenting *Vibrio* spp. were detected at lower levels of 3.2 ± 0.4 and 3.4 ± 0.5 log CFU/g, respectively (Figure 1A). The predominance of *Shewanella* spp. suggested a high spoilage potential because these bacteria are recognized as specific spoilage organisms capable of producing off-odor sulfur-containing compounds in seafood during chilled storage [29,30]. The occurrence of *Aeromonas* spp. and *Vibrio* spp. reflected that the samples encountered environmental and marine-associated contamination. The detection of *V. parahaemolyticus*, an important seafood-borne pathogen, highlighted potential microbiological safety concerns associated with crossed contamination as well as inadequate cooking of shellfish products [20].

A total of 102 bacterial isolates were obtained from 25 samples using selective media targeting *Vibrio*, *Shewanella*, and *Aeromonas* groups, and species identification was carried out using MALDI-TOF MS (Figure 1B,C). The isolates were dominated by *V. parahaemolyticus*, *V. alginolyticus*, *V. harveyi*, *S. indica*, *S. algae*, and several *Aeromonas* species, including *A. hydrophila*, *A. veronii*, *A. bestiarum*, and *A. eucrenophila*. The high diversity of Gram-negative marine bacteria indicated that RAMs harbored a complex microbiota as influenced by environmental exposure, post-processing contamination, and improper refrigerated storage conditions. The coexistence of *Aeromonas* and *Vibrio* may reflect differences in environmental salinity, while the presence of spoilage-associated and pathogenic bacteria raises the concerns regarding product quality and consumer safety [31].

Among the isolates, *V. parahaemolyticus* had the largest proportion (26.5%), suggesting that the seafood matrix favored the growth of halophilic marine bacteria. In addition to its role in spoilage, *V. parahaemolyticus* is a well-known causative microorganism of seafood-associated gastroenteritis. The detection of *V. alginolyticus*, *V. harveyi*, and *V. fluvialis* further demonstrated the complexity of the marine bacterial community contaminated in shellfish products. Interestingly, several isolates presumptively identified as *Vibrio* spp. on TCBS agar were subsequently identified as non-*Vibrio* bacteria, including *S. algae*, *S. indica*, *Proteus hauseri*, *Exiguobacterium* sp., and *A. veronii*. This finding demonstrated the limited selectivity of TCBS agar when applied to complex seafood microbiota and highlighted the importance of MALDI-TOF MS for accurate bacterial identification [20,21,32]. The recovery of non-target bacteria on TCBS agar also suggested that some seafood-associated bacteria possess physiological tolerance to bile salts and alkaline conditions [33].

The antimicrobial susceptibility results demonstrated that CEGC exhibited stronger antibacterial activity than nisin against the tested seafood-associated bacteria (Supplementary Table S1). The minimum inhibitory concentration (MIC) values of CEGC ranged from 156 to 625 µg/mL, whereas nisin generally showed much higher MIC values (2500–5000 µg/mL). The lowest MIC (156 µg/mL) of CEGC was mainly observed in *V. parahaemolyticus* and one isolate of *V. alginolyticus*, indicating high susceptibility of *Vibrio* spp. to CEGC. This enhanced sensitivity might be mediated by membrane destabilization and oxidative stress induced by EGCG conjugated with COS [13].

In contrast, comparatively higher MIC values against both CEGC and nisin were found for *Aeromonas* spp. Several isolates of *A. hydrophila*, *A. bestiarum*, and *A. eucrenophila* required 5000 µg/mL nisin for their inhibition, indicating strong intrinsic resistance of those microorganisms to both compounds tested. The low susceptibility of *Aeromonas* spp. might be related to the protective outer membrane barrier and stress-response mechanisms characteristic of Gram-negative bacteria. Similarly, *Shewanella* isolates showed intermediate susceptibility to CEGC, with MIC values ranging from 313 to 625 µg/mL. Since *Shewanella* spp. are major spoilage bacteria responsible for sulfurous off-odor and trimethylamine production, the inhibition of these bacteria is technologically important for extending shelf life of seafood [11,34]. Species-dependent differences in antimicrobial susceptibility were also observed. *V. parahaemolyticus* isolates exhibited MIC values of 156–313 µg/mL, whereas *V. harveyi* and *V. anguillarum* generally required higher concentrations (313–625 µg/mL). Such variability plausibly reflected the differences in membrane composition, stress adaptation, quorum sensing, or biofilm-forming ability among marine bacterial species [35]. Notably, the antimicrobial profiles of standard strains were comparable to those of environmental isolates. The MIC of CEGC toward standard strains, including *V. parahaemolyticus*, *V. alginolyticus*, *A. hydrophila*, *A. veronii*, and *S. putrefaciens* was approximately 313 µg/mL. Conversely, MIC of nisin against those bacteria was in the range of 2500–5000 µg/mL. The result revealed broad-spectrum antibacterial activity of CEGC, while nisin had the lower antimicrobial activity.

The minimum bactericidal concentration (MBC) results further confirmed the superior antibacterial efficacy of CEGC, compared to nisin (Supplementary Table S1). MBC of CEGC toward *Vibrio* spp. isolates, particularly *V. parahaemolyticus*, was ranged from 625 to 1250 µg/mL. Similar to MIC, nisin showed high MBC, reflecting substantially higher concentrations (2500–5000 µg/mL) required to achieve bactericidal effects. The relatively low MBC values suggested that CEGC exerted not only growth inhibition but also efficient bacterial killing. Interestingly, several *Shewanella* and *Aeromonas* isolates showed identical MIC and MBC values when CEGC was used, indicating rapid bactericidal action when the inhibitory concentration was reached. In contrast, nisin frequently exhibited markedly higher MBC values than its MIC values, particularly among *Aeromonas* spp., reflecting weaker killing efficiency against Gram-negative bacteria. These findings suggested that CEGC likely induces the irreversible membrane damage and increased cellular permeability, leading to the enhanced bacterial inactivation.

Overall, RAMs contained diverse Gram-negative marine bacteria associated with both spoilage and foodborne risk. CEGC exhibited substantially stronger antibacterial activity than nisin, particularly against *Vibrio* and *Shewanella* spp., highlighting its potential as a multifunctional natural preservative for seafood preservation and for ensuring the safety.

### 3.2. Characterization of Virulence-Associated Phenotypes

**Figure 2:**
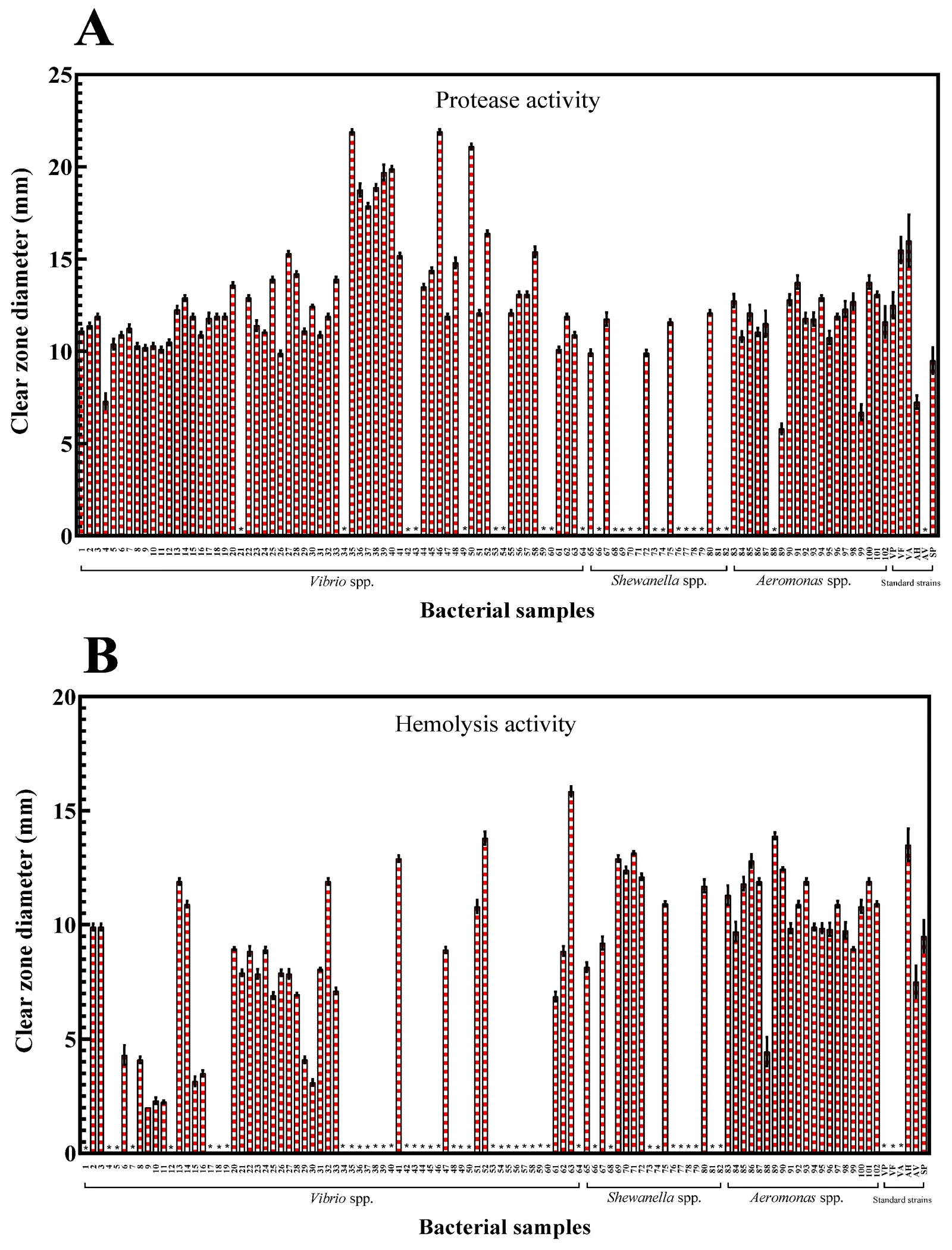
Extracellular protease production and hemolytic activity of seafood-associated bacterial isolates. Representative images showing extracellular protease activity (**A**) and hemolysis on blood agar (**B**) of 102 isolates of *Vibrio* spp., *Shewanella* spp., and *Aeromonas* spp. after incubation at 37 °C for 24 h. The diameters of the clear zones were measured to evaluate extracellular enzymatic and hemolytic activities. Vertical bars represent the standard deviation of triplicate determinations (*n* = 3). An asterisk (*) indicates no detectable extracellular protease activity. Presumptive isolates included *Vibrio* spp. (isolate nos. 1–64), *Shewanella* spp. (isolate nos. 65–82), and *Aeromonas* spp. (isolate nos. 83–102). Reference strains included *Vibrio parahaemolyticus* ATCC 17802, *Vibrio fluvialis* BL186, *Vibrio alginolyticus* DMST 14800, *Aeromonas hydrophila* ATCC 7966, *Aeromonas veronii* ATCC 35624, and *Shewanella putrefaciens* JCM 20190.

Representative bacterial isolates confirmed by MALDI-TOF/MS exhibited diverse virulence-associated phenotypes, particularly proteolytic and hemolytic activities. The isolates mainly belonging to *Vibrio*, *Aeromonas*, and *Shewanella* species, are commonly associated with marine environments and seafood products [10]. Proteolytic activity was broadly detected among most isolates, especially *V. alginolyticus*, *V. harveyi*, *Aeromonas* spp., and several *Shewanella* isolates (Figure 2A). Some *V. alginolyticus* isolates showed the strongest protease production, with clear zone diameters exceeding 18–21 mm, although no hemolytic activity was observed (Figure 2B). This finding suggested that certain *Vibrio* species might contribute to seafood spoilage rather than pathogenicity through the secretion of extracellular proteases. These enzymes can accelerate muscle protein degradation, leading to texture softening and accumulation of spoilage-related compounds during refrigerated storage [36]. In contrast, several *Aeromonas* isolates exhibited both proteolytic and hemolytic activities. *A. hydrophila*, *A. veronii*, and *A. bestiarum* showed pronounced hemolysis together with moderate-to-high protease activity, indicating greater virulence potential than many *Vibrio* isolates. This observation reconfirmed that *Aeromonas* spp. are recognized as opportunistic foodborne pathogens frequently associated with minimally processed seafood products [21].

The β-hemolytic phenotype of *Aeromonas* spp. is closely associated with the production of hemolysins, including aerolysin and cytotoxic enterotoxins, which play the important role in pathogenicity during human infection [37]. Interestingly, different hemolytic activity was observed among the isolates recovered from refrigerated samples, which might be associated with the storage condition and/or inherent variation among isolates. Similar stress-induced responses have been reported in seafood-associated bacteria exposed to low-temperature storage [20,38]. These findings highlighted potential health risks caused by contaminated RAM, particularly when products are improperly handled or consumed raw or undercooked. Previous studies have also demonstrated that fresh and refrigerated clams harbor diverse *Aeromonas* species, especially *A. hydrophila* and *A. veronii*, exhibiting hemolytic activity together with spoilage-associated traits, thereby emphasizing their dual roles in seafood deterioration and foodborne pathogenicity [20]. However, the underlying mechanisms remain unclear, and further studies involving controlled storage experiments with the same strains and gene expression analysis are needed to determine whether cold stress influences the expression of hemolysin- and other virulence-related genes.

Considerable phenotypic variation was observed even within the same bacterial species. Some *V. parahaemolyticus* isolates demonstrated hemolytic activity, whereas others showed only proteolytic activity or phenotype. Such variability was probably related to strain-dependent regulation of virulence genes and environmental adaptation. Therefore, species identification alone might not accurately reflect pathogenic potential, thus emphasizing the importance of combining phenotypic characterization with MALDI-TOF/MS identification. *Shewanella* isolates also exhibited variable phenotypes. Certain isolates identified as *S. indica* and *S. algae* displayed strong hemolytic activity but weak proteolytic activity, while others showed the opposite pattern. Although *Shewanella* spp. are mainly recognized as seafood spoilage bacteria associated with sulfurous off-odor and trimethylamine production, the present findings indicated that some strains might also possess opportunistic virulence-associated traits [11]. Another notable observation was the discrepancy between presumptive identification on selective media and MALDI-TOF/MS confirmation. Several isolates obtained from *Vibrio*-selective agar were ultimately identified as *Aeromonas*, *Shewanella*, *Proteus*, or *Exiguobacterium*. This result highlighted the limited specificity of selective media when applied to complex seafood microbiota. Thus, MALDI-TOF/MS was still required for accurate bacterial identification. Overall, RAM contained a complex bacterial community comprising both spoilage-associated and potentially opportunistic pathogenic bacteria. The presence of proteolytic and hemolytic activities among the isolates not only resulted in rapid quality deterioration but also increased food safety concerns of RAM during storage and distribution.

**Figure 3:**
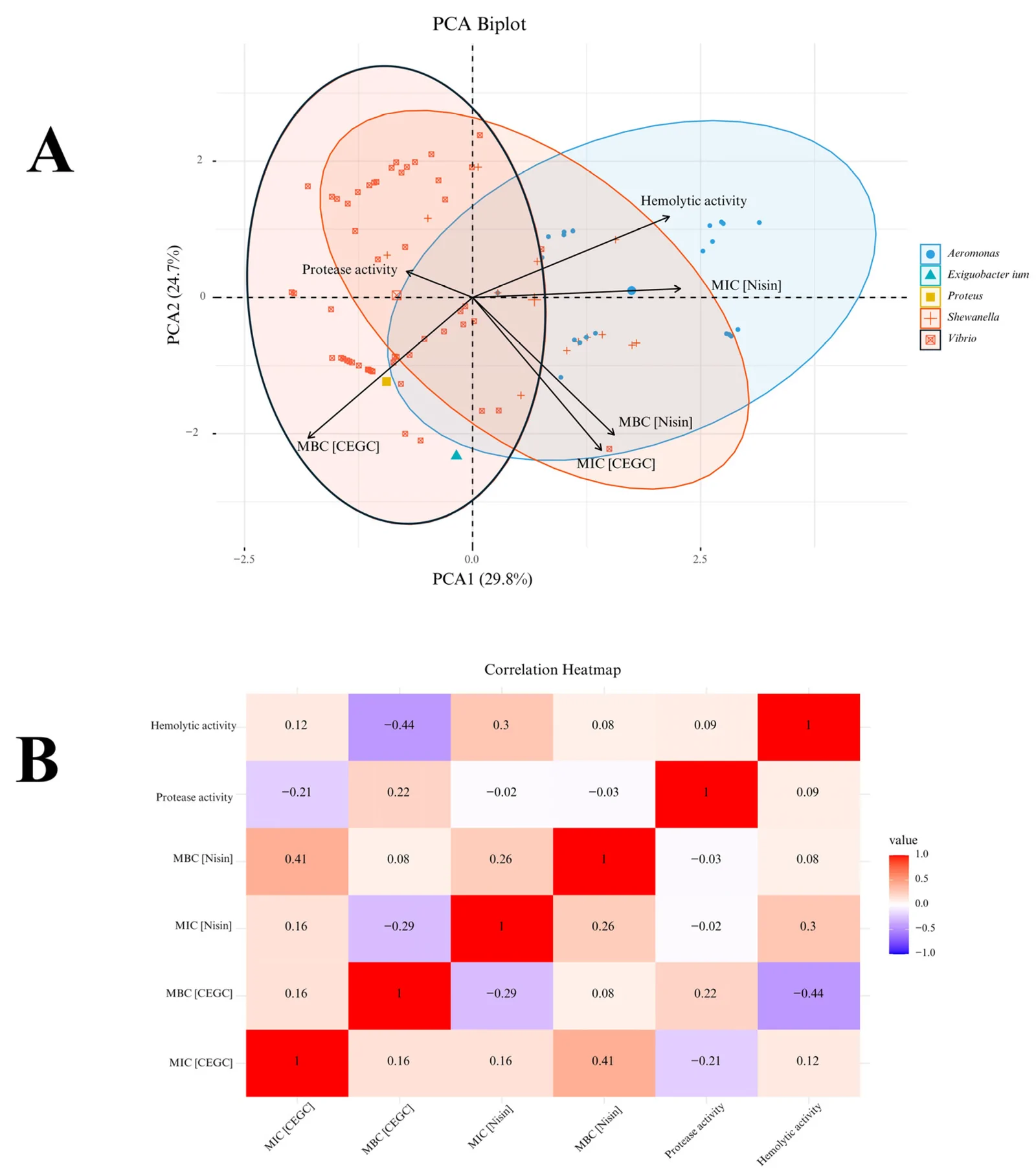
Principal component analysis (PCA) and Pearson correlation heatmap showing the relationships between antimicrobial susceptibility (MIC and MBC of CEGC and nisin) and virulence-associated phenotypes (hemolytic and proteolytic activities) of 102 seafood-associated bacterial isolates identified by MALDI-TOF/MS. (**A**) PCA biplot and (**B**) Pearson correlation matrix heatmap. Blue circles represent *Aeromonas* spp., orange circles represent *Shewanella* spp., and black circles represent *Vibrio* spp.

Principal component analysis (PCA) was performed to integrate antimicrobial susceptibility (MIC and MBC of CEGC and nisin) with virulence-associated phenotypes (hemolytic and proteolytic activities) of the 102 seafood-associated bacterial isolates (Figure 3A). The first two principal components explained 54.5% of the total variance (PC1 = 29.8% and PC2 = 24.7%), indicating that these variables captured the major phenotypic variation among the isolates. The loading plot showed that MIC and MBC values of each antimicrobial were closely aligned, reflecting the consistency between inhibitory and bactericidal activities. In contrast, hemolytic and proteolytic activities were projected in different directions, indicating that these virulence-associated traits contributed independently to the overall phenotypic variation. The score plot revealed considerable phenotypic heterogeneity among the bacterial isolates. Although partial clustering by bacterial genus was observed, substantial overlap among *Vibrio*, *Aeromonas*, and *Shewanella* indicated that antimicrobial susceptibility and virulence-associated phenotypes varied among strains within the same genus rather than being strictly genus-dependent. The correlation heatmap further demonstrated generally weak-to-moderate relationships among the evaluated variables (Figure 3B). MIC and MBC values of the same antimicrobial were positively correlated, whereas hemolytic and proteolytic activities showed only weak associations with antimicrobial susceptibility. These results suggested that bacterial susceptibility to CEGC and nisin was largely independent of extracellular virulence-associated phenotypes. The multivariate analysis complemented the findings presented by demonstrating that the observed diversity in antimicrobial susceptibility and virulence characteristics was primarily strain-dependent. Together, these results highlighted the phenotypic complexity of seafood-associated bacterial communities and emphasized the importance of evaluating antimicrobial susceptibility and virulence traits simultaneously when assessing the preservation efficacy of natural antimicrobial agents.

### 3.3. Minimum Inhibitory Concentration (MIC) and Fractional Inhibitory Concentration Index (FICI) of Antimicrobial Agent Against Seafood-Associated Bacteria

The antibacterial activities of CEGC and nisin against the selected seafood-associated bacteria were evaluated using MIC and FICI (Table 1). CEGC showed consistent antibacterial activity against all tested strains, with MIC values of 313 µg/mL. In contrast, nisin exhibited weaker and strain-dependent activity, with MIC values ranging from 2500 to 5000 µg/mL. *V. parahaemolyticus*, *V. alginolyticus*, *S. putrefaciens*, and *A. veronii* were more susceptible to nisin (2500 µg/mL), whereas *V. fluvialis* and *A. hydrophila* required higher concentrations (5000 µg/mL). The checkerboard assay demonstrated that combining CEGC with nisin enhanced antibacterial activity against all tested bacteria. Synergistic effects (FICI = 0.50) were observed against *V. parahaemolyticus* and *V. fluvialis*, whereas additive effects (FICI = 1.00) were observed against *V. alginolyticus*, *S. putrefaciens*, and *A. veronii*. In contrast, an indifferent effect (FICI = 1.06) was observed against *A. hydrophila*. The combined treatment reduced the MICs of CEGC and nisin; however, the extent of reduction varied among bacterial strains. The results indicated that CEGC possessed stronger antibacterial activity than nisin alone against Gram-negative seafood-associated bacteria. The enhanced efficacy of CEGC might be related to the combined properties of COS and EGCG, including improved solubility, increased cationic charge density, and membrane-disruptive activity [39,40]. These characteristics likely promoted the interactions with the bacterial outer membrane, resulting in increased permeability and cellular damage. Nisin alone is generally less effective against Gram-negative bacteria because the outer membrane restricts its access to the cytoplasmic membrane [41]. Previous studies also showed that the MIC of nisin against *Listeria monocytogenes* ATCC 19115 ranged from 50 to 200 μg/mL at pH 3.2 to 7.0 [42]. However, the improved activity observed in the combined treatments suggested that CEGC facilitated nisin penetration by destabilizing the outer membrane. This mechanism might explain the synergistic effects observed particularly in *V. parahaemolyticus* and *V. fluvialis*. When the permeability barrier is disrupted, nisin can more effectively interact with the cytoplasmic membrane and induce pore formation [43]. Although only additive effects were observed in some strains, the reduction in MIC values still demonstrated the benefit of combined treatments. Lower antimicrobial concentrations might help reduce the adverse impact on sensory property and lower the amount of preservative residue retained in seafood. The inhibitory effects against *Vibrio*, *Aeromonas*, and *Shewanella* species are particularly crucial because these bacteria are closely associated with seafood spoilage and foodborne illness. Therefore, the combined use of CEGC and nisin could provide an effective natural preservation approach for improving the microbial safety and extending the shelf life of RAM products.

### 3.4. Inactivation of Seafood-Associated Bacteria by CEGC and Nisin

**Figure 4:**
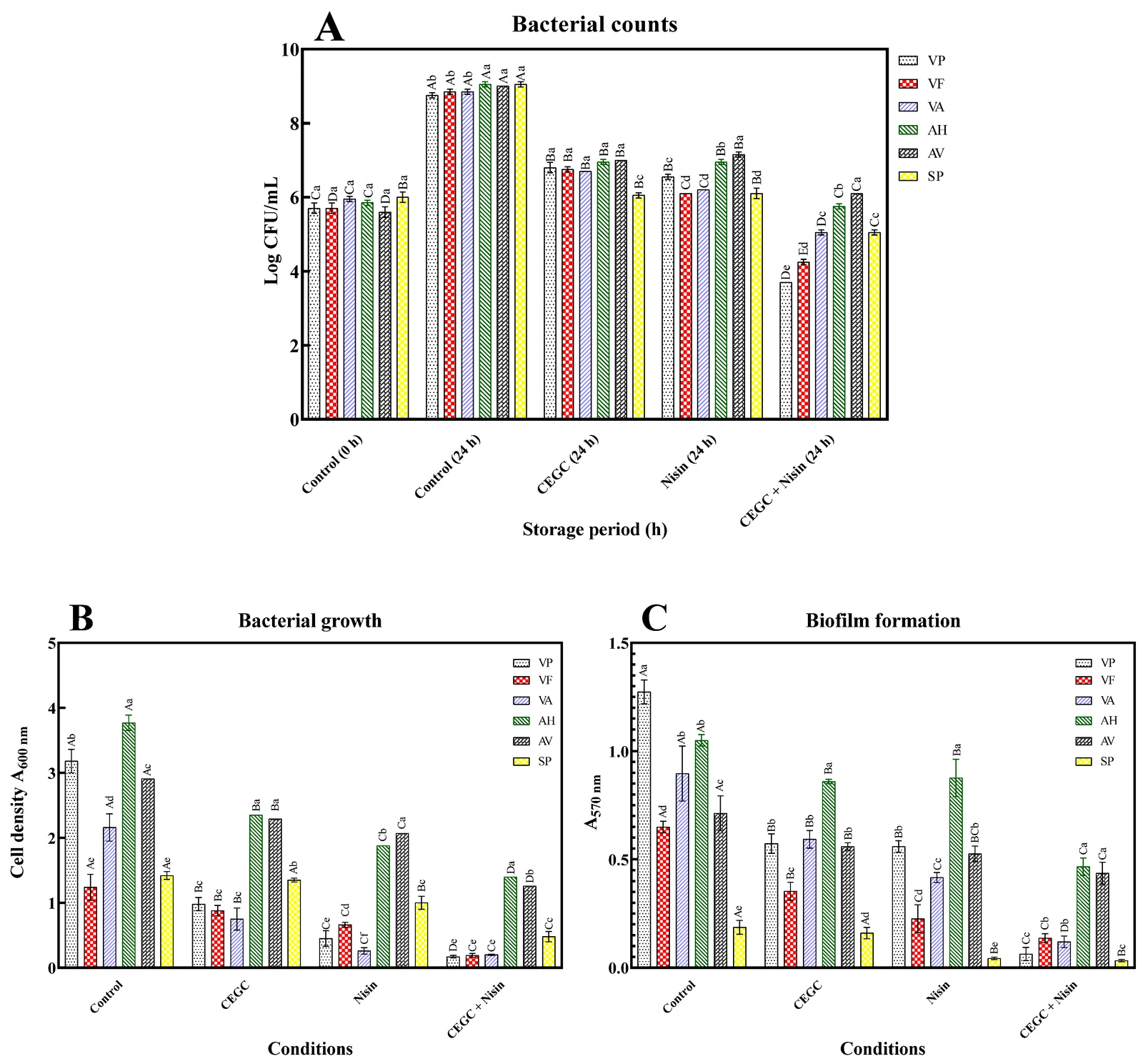
Antibacterial, growth inhibitory, and antibiofilm activities of chitooligosaccharide–epigallocatechin gallate conjugate (CEGC), nisin, and their combination against seafood-associated pathogenic and spoilage bacteria. (**A**) Bacterial inactivation after 24 h of treatment. (**B**) Bacterial growth inhibition. (**C**) Biofilm formation after treatment with CEGC, nisin, and their combined treatments. Tested bacterial strains included *Vibrio parahaemolyticus* ATCC 17802 (VP), *Vibrio fluvialis* BL186 (VF), *Vibrio alginolyticus* DMST 14800 (VA), *Aeromonas hydrophila* ATCC 7966 (AH), *Aeromonas veronii* ATCC 35624 (AV), and *Shewanella putrefaciens* JCM 20190 (SP). Values are presented as mean ± standard deviation (SD) from three independent experiments (*n* = 3). Different uppercase letters on the bars indicate significant differences among treatments within the same bacterial strain, whereas different lowercase letters on the bars indicate significant differences among bacterial strains within the same treatment (*p* < 0.05).

The antibacterial activities of CEGC, nisin, and their combination against seafood-associated bacteria were evaluated after 24 h of treatment. In the untreated control, all tested bacteria rapidly proliferated from initial populations of approximately 5.60–6.00 log CFU/mL to 8.75–9.05 log CFU/mL, indicating high growth capacity under the tested conditions (Figure 4A). CEGC alone (313 µg/mL) effectively inhibited bacterial growth, resulting in the final counts of 6.05–7.00 log CFU/mL after 24 h. Nisin alone (2500 µg/mL) also reduced bacterial populations, although its activity varied among bacterial species. *V. fluvialis* and *V. alginolyticus* were relatively more susceptible to nisin, whereas *A. veronii* showed greater tolerance. The combined treatments were applied at strain-specific combined MIC concentrations determined by the checkerboard assay (Table 1). These concentrations ranged from 78 to 313 µg/mL for CEGC and from 313 to 1250 µg/mL for nisin. The strongest antibacterial effects were observed for the combined treatments, with the magnitude of inhibition varying among bacterial strains. Despite the substantially lower concentrations, the combination treatments markedly suppressed bacterial growth. Populations of *V. parahaemolyticus*, *V. fluvialis*, and *V. alginolyticus* decreased to 3.70, 4.25, and 5.05 log CFU/mL, respectively, corresponding to reductions of approximately 5.05, 4.60, and 3.80 log CFU/mL, compared with the untreated control. Similarly, *A. hydrophila*, *A. veronii*, and *S. putrefaciens* were reduced to 5.75, 6.10, and 5.05 log CFU/mL, respectively. These results confirmed that the combined treatments exerted stronger antibacterial activity than individual antimicrobial agents.

The rapid proliferation observed in the control groups reflected the strong adaptability of *Vibrio*, *Aeromonas*, and *Shewanella* species, which are commonly associated with seafood spoilage and foodborne illness. CEGC alone showed considerable antibacterial activity, likely due to the combined effects of COS and EGCG. The cationic properties of COS and the phenolic structure of EGCG could disrupt membrane integrity, increase membrane permeability, and interfere with essential cellular functions. Although nisin is generally less effective against Gram-negative bacteria because of the protective outer membrane, the moderate inhibitory effects were still observed. The enhanced antibacterial activity in the combined treatments suggested that CEGC facilitated nisin penetration by destabilizing the outer membrane, allowing nisin to interact more effectively with the cytoplasmic membrane and induce pore formation [14,44,45]. The synergistic effect was particularly evident in *Vibrio* species, which appeared more susceptible than *A. veronii*. This difference could be related to species-specific membrane composition and variation in stress-response mechanisms. Importantly, the combined treatments achieved substantial bacterial reductions at lower concentrations than those required for the individual antimicrobial treatments, although the magnitude of inhibition varied among bacterial strains.

### 3.5. Effect of CEGC and Nisin on Bacterial Growth and Biofilm Formation

The effects of CEGC, nisin, and their combination on bacterial growth and biofilm formation were evaluated after 24 h of incubation (Figure 4B,C). In untreated controls, all tested bacteria showed substantial growth, with OD_600_ values ranging from 1.24 to 3.77. *A. hydrophila* and *V. parahaemolyticus* exhibited the highest cell densities, indicating strong proliferation under the experimental conditions. CEGC alone significantly inhibited bacterial growth in all tested strains, reducing OD_600_ values to 0.75–2.35. Nisin alone showed stronger inhibition against *Vibrio* species, particularly *V. parahaemolyticus*, *V. fluvialis*, and *V. alginolyticus*, whose OD_600_ values decreased to 0.45, 0.66, and 0.26, respectively. In contrast, *Aeromonas* species were less sensitive to the individual treatments. The combined treatments, applied at the respective strain-specific combined MIC concentrations as listed in Table 1, produced the strongest inhibitory effects against all tested bacteria. OD_600_ values of *V. parahaemolyticus*, *V. fluvialis*, and *V. alginolyticus* decreased to 0.17, 0.19, and 0.20, respectively, corresponding to more than 90% reduction, compared with that observed in untreated controls. Similar inhibitory effects were observed in *A. hydrophila*, *A. veronii*, and *S. putrefaciens*.

A similar trend was observed for biofilm formation (Figure 4C). Untreated bacteria produced measurable biofilms with OD_570_ values ranging from 0.19 to 1.27, with *V. parahaemolyticus* showing the highest biofilm-forming ability. Treatment with CEGC or nisin alone moderately reduced biofilm formation, whereas the combined treatments at the respective strain-specific combined MIC concentrations showed the greatest antibiofilm activity. Biofilm biomass of *V. parahaemolyticus* decreased from 1.27 in the control to 0.06 after combined treatments, representing approximately 95% inhibition. Strong reductions were also observed in *V. fluvialis* and *V. alginolyticus*, whose OD_570_ values decreased to 0.14 and 0.12, respectively. Biofilm formation in *Aeromonas* and *Shewanella* species was also significantly reduced, although the effect was less pronounced than that found in *Vibrio* spp. The results demonstrated that the combination of CEGC and nisin effectively suppressed both planktonic growth and biofilm formation of seafood-associated bacteria. Biofilms are important for bacterial survival because they enhance resistance to environmental stress, antimicrobial agents, and sanitation processes [46]. In seafood-processing environments, biofilm-forming bacteria such as *Vibrio*, *Aeromonas*, and *Shewanella* contribute to persistent contamination and accelerated spoilage [47].

The antibacterial activity of CEGC might be governed by the polycationic properties of COS and the membrane-disruptive effects of EGCG. These compounds were able to alter membrane permeability, induce oxidative stress, and interfere with essential cellular processes [48]. In addition, EGCG, a catechin derivative, might affect quorum sensing and extracellular polymeric substance production, both of which are important for biofilm development [49].

Nisin alone also inhibited bacterial growth and biofilm formation, particularly in *Vibrio* species. However, its activity against Gram-negative bacteria was generally limited by the outer membrane barrier. The enhanced effect observed in the combined treatment suggested that CEGC increased membrane permeability, thereby facilitating nisin penetration into bacterial cells. Among the tested bacteria, *V. parahaemolyticus* exhibited the highest susceptibility to the combined treatment, in which the growth and biofilm formation were inhibited to the highest extent. Overall, the synergistic combination of CEGC and nisin effectively inhibited bacterial growth and biofilm formation at relatively low concentrations, compared with individual agents.

### 3.6. Effect of CEGC and Nisin on Cell Membrane Permeability and Morphology

**Figure 5:**
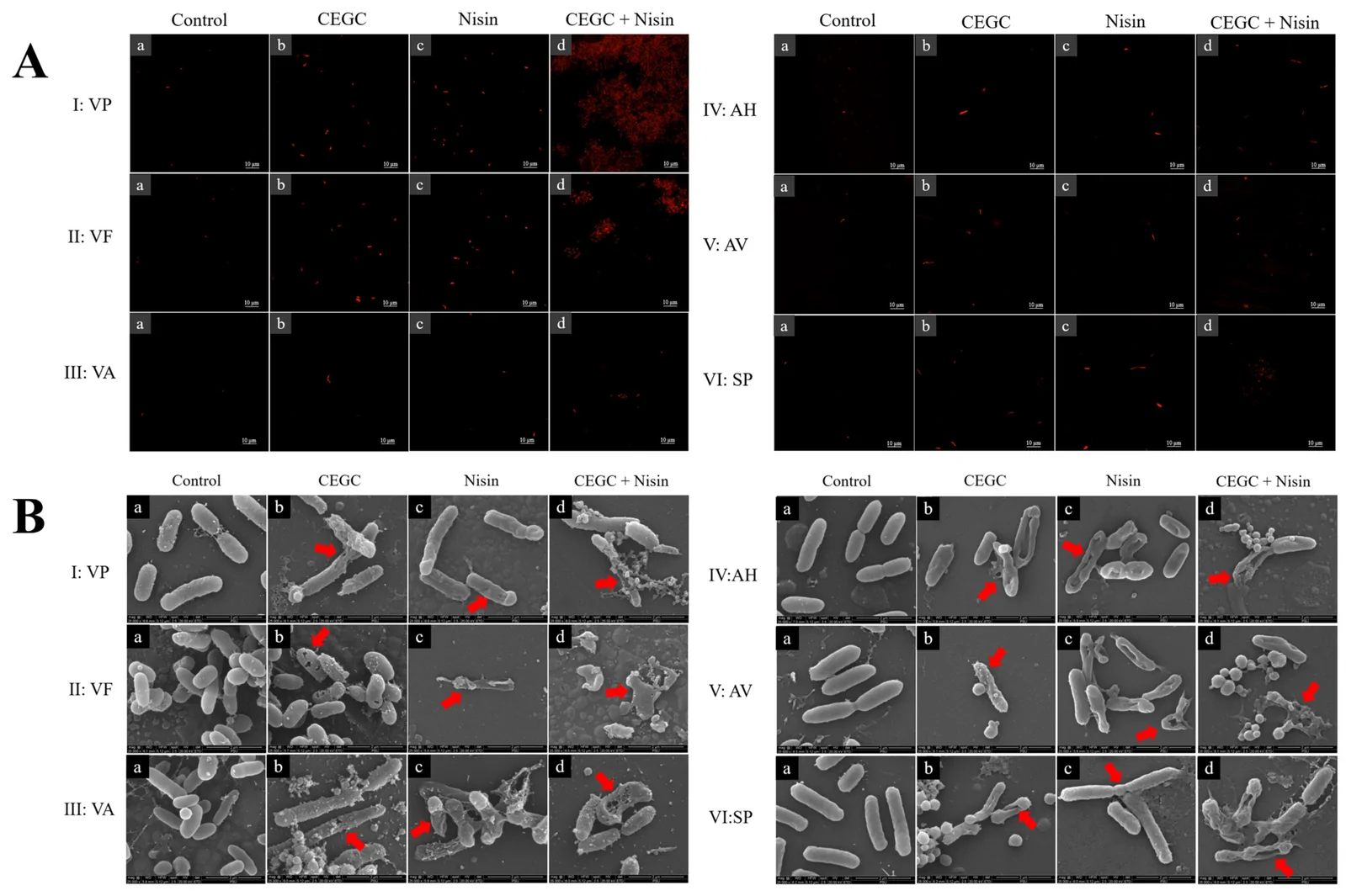
Effects of chitooligosaccharide–epigallocatechin gallate conjugate (CEGC), nisin, and their synergistic combination on bacterial cell membrane integrity and morphology. (**A**) Cell membrane permeability of treated bacterial cells. (**B**) Scanning electron microscopic (SEM) images of untreated control cells (**a**) and cells treated with CEGC (**b**), nisin (**c**), and the synergistic combination of CEGC and nisin (**d**). Tested bacterial strains included *Vibrio parahaemolyticus* ATCC 17802 (**I: VP**), *Vibrio fluvialis* BL186 (**II: VF**), *Vibrio alginolyticus* DMST 14800 (**III: VA**), *Aeromonas hydrophila* ATCC 7966 (**IV: AH**), *Aeromonas veronii* ATCC 35624 (**V: AV**), and *Shewanella putrefaciens* JCM 20190 (**VI: SP**). SEM observations were performed at a magnification of 30,000×. Arrows indicate the damaged or morphologically altered bacterial cells following antimicrobial treatment.

Membrane damage in seafood-associated bacteria, including *V. parahaemolyticus* (VP), *V. fluvialis* (VF), *V. alginolyticus* (VA), *A. hydrophila* (AH), *A. veronii* (AV), and *S. putrefaciens* (SP), was evaluated using propidium iodide (PI) fluorescence staining and scanning electron microscopy (SEM) (Figure 5). Untreated cells showed weak fluorescence signals, indicating intact membrane integrity and low permeability of PI (Figure 5(a)). In contrast, cells treated with CEGC, nisin, and especially their combination exhibited strong red fluorescence, indicating severe membrane disruption and increased permeability (Figure 5A(b–d)). The combined treatment produced the highest fluorescence intensity, even when the combined antimicrobial agents were used at lower concentrations. CEGC and nisin were applied at 78–156 µg/mL and 625–1250 µg/mL, respectively, whereas single treatments required higher concentrations of CEGC (313 µg/mL) and nisin (2500–5000 µg/mL). The strongest membrane permeability effects were observed in *V. parahaemolyticus* and *V. fluvialis*, which also showed synergistic interactions in the checkerboard assay (FICI = 0.50) (Figure 5A(I–II(d))). Although *V. alginolyticus*, *A. hydrophila*, *A. veronii*, and *S. putrefaciens* showed the additive effects (FICI = 1.00), substantial membrane damage was still evident (Figure 5A(III–VI(d))). The membrane-disruptive effect of CEGC may be attributed to the physicochemical properties of EGCG, which can interact with membrane lipids and proteins, thereby affecting membrane integrity [46]. Nisin alone also increased membrane permeability, although its effect was less pronounced [50]. Since Gram-negative bacteria possess an outer membrane barrier that limits nisin penetration [51], the stronger effect observed in the combined treatment suggested that CEGC increased membrane permeability and promoted nisin entry into the cytoplasmic membrane, where pore formation and membrane depolarization occurred [46,52].

SEM observations supported the fluorescence results (Figure 5B). Untreated cells maintained normal rod-shaped morphology with smooth and intact surfaces (Figure 5B(a)). Cells treated with CEGC alone showed membrane shrinkage, deformation, pore formation, and surface wrinkling (Figure 5B(b)). Similar alterations were observed in nisin-treated cells, although structural damage was less extensive (Figure 5B(c)). The combined treatments caused the most severe morphological destruction (Figure 5B(d)). Cells exhibited an extensive membrane rupture, deep surface collapse, cellular fragmentation, and leakage of intracellular contents [14,53]. In several micrographs, bacterial cells lost their recognizable structure entirely, indicating irreversible membrane damage and cell death. The strongest effects were observed in *Vibrio* species, particularly *V. parahaemolyticus* and *V. fluvialis* (Figure 5B(I–II(d))). These findings suggested that membrane disruption was a major antibacterial mechanism underlying the synergistic interaction between CEGC and nisin. The extensive ultrastructural damage observed under combined treatment strongly supported this complementary mode of action. Similar findings were reported in *Escherichia coli* O157 treated with a combination of nisin and phytic acid, where severe membrane disruption and extensive cell lysis were observed [50]. Overall, the combination of CEGC and nisin effectively damaged bacterial membranes and caused extensive ultrastructural destruction in seafood-associated bacteria.

### 3.7. Changes in Biofilm Formation

**Figure 6:**
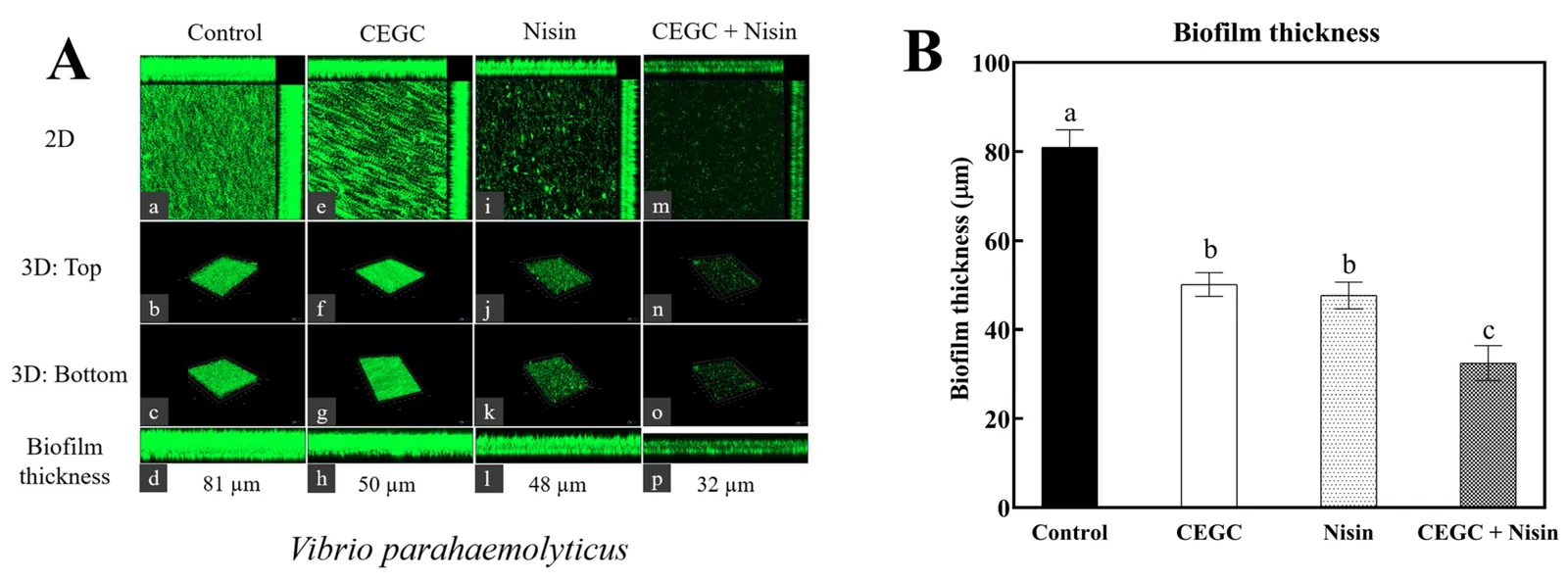
Confocal laser scanning microscopic (CLSM) analysis of *Vibrio parahaemolyticus* ATCC 17802 biofilms after treatment with chitooligosaccharide–epigallocatechin gallate conjugate (CEGC), nisin, and their synergistic combination. (**A**) Representative CLSM images showing two-dimensional (2D) images (**a**,**e**,**i**,**m**), three-dimensional top-view images (**b**,**f**,**j**,**n**), three-dimensional bottom-view images (**c**,**g**,**k**,**o**), and z-stack reconstructed images for biofilm thickness analysis (**d**,**h**,**l**,**p**) of live bacterial biofilms formed on 4-well cell culture chamber slides. Scale bar = 50 μm. (**B**) Quantitative analysis of biofilm thickness after antimicrobial treatments. Values are expressed as mean ± standard deviation (SD) from three independent experiments (*n* = 3). Different lowercase letters on the bars indicate significant differences among various treatments at the same minimum inhibitory concentration (MIC) level (*p* < 0.05).

*V. parahaemolyticus* was selected for confocal laser scanning microscopic (CLSM) analysis because it exhibited strong biofilm-forming ability and high susceptibility to the combined treatment of CEGC and nisin (Figure 6A). This species is an important seafood-associated pathogen capable of forming robust biofilms that enhance environmental persistence, antimicrobial resistance, and surface attachment [54]. CLSM analysis using SYTO9 staining was performed to evaluate the effects of CEGC, nisin, and their combination on biofilm formation and architecture (Figure 6A). Untreated biofilms exhibited dense and well-organized structures with strong green fluorescence, indicating a high abundance and dense distribution of SYTO 9-stained bacterial cells within the biofilm. In contrast, treatment with CEGC or nisin alone reduced fluorescence intensity and alterations in biofilm organization. Biofilms treated with CEGC appeared less compact, whereas nisin treatment reduced cell density and partially disrupted biofilm structure. The combined treatment exhibited the strongest antibiofilm effect, as evidenced by sparse bacterial distribution, disrupted biofilm architecture, and markedly lower fluorescence intensity. These observations indicated substantial changes in biofilm distribution and structural organization after the combined treatment. Quantitative analysis further confirmed the CLSM observations (Figure 6B). The untreated control exhibited the highest biofilm thickness (81.0 ± 3.9 µm), whereas treatment with CEGC and nisin alone reduced thickness to 50.1 ± 2.7 and 47.7 ± 3.0 µm, respectively. The combined treatments reduced biofilm thickness to 32.5 ± 4.0 µm, corresponding to approximately 60.5% reduction compared with that of the control. This result was consistent with the synergistic antibacterial interaction observed in the checkerboard assay (Table 1). The strong antibiofilm activity of the combined treatments plausibly involved multiple complementary mechanisms. CEGC likely interfered with membrane integrity, bacterial adhesion, and extracellular polymeric substance (EPS) stability. In addition, EGCG possibly affected quorum sensing and extracellular matrix production, which are essential for biofilm maturation [55]. Generally, biofilms formed by *V. parahaemolyticus* can promote bacterial persistence and increase the risk of cross-contamination during processing and storage. The ability of the CEGC–nisin combination to reduce biofilm biomass and viable biofilm cells showed the promising approach for controlling seafood-associated pathogens in food systems. Collectively, the CLSM observations demonstrated that the synergistic combination of CEGC and nisin effectively disrupted biofilm structure, reduced viable bacterial populations, and inhibited biofilm maturation in *V. parahaemolyticus*.

### 3.8. Antimicrobial Activity of CEGC and/or Nisin Against Vibrio parahaemolyticus in Refrigerated RAM

**Figure 7:**
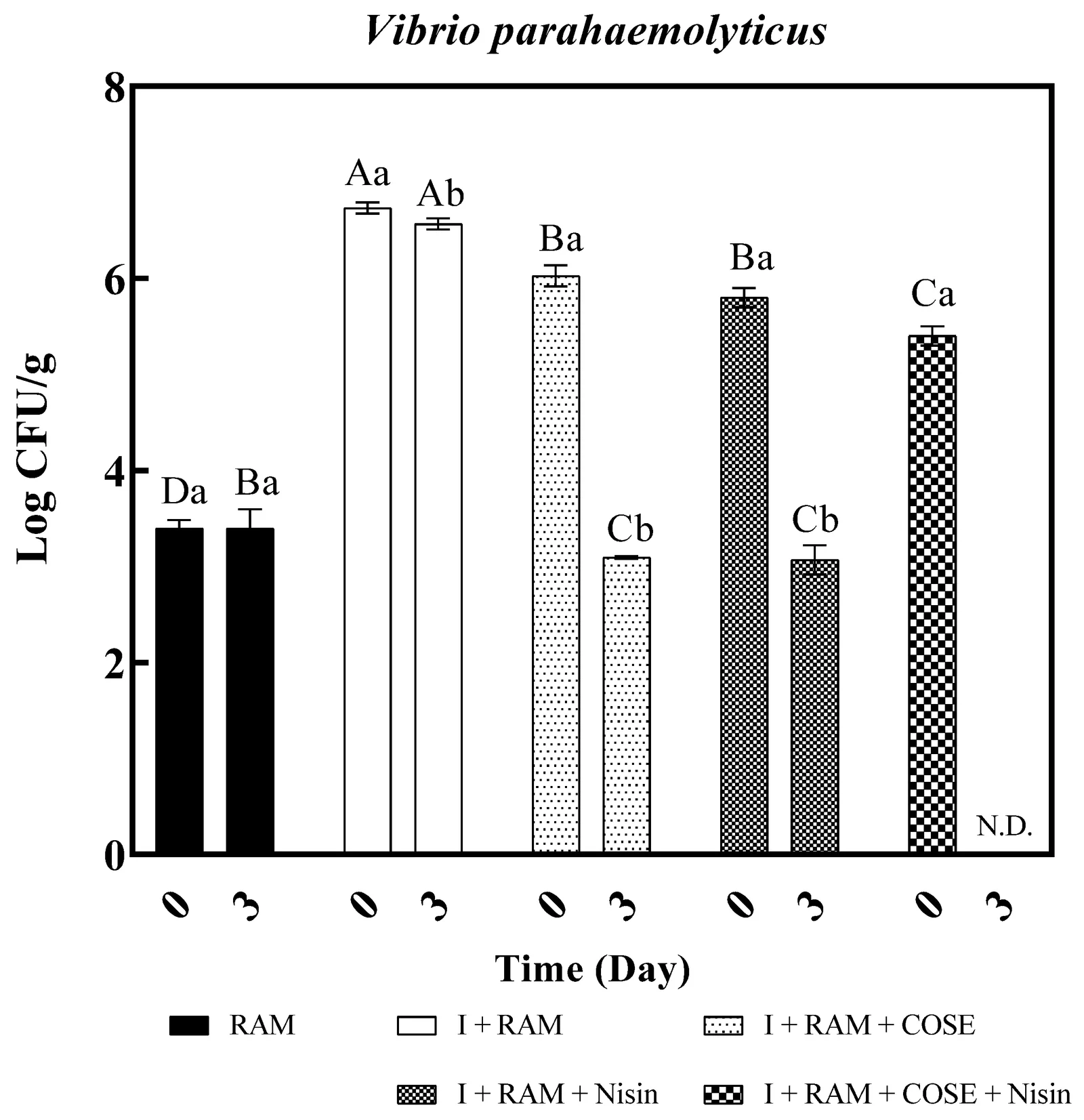
Changes in *Vibrio parahaemolyticus* ATCC 17802 populations in inoculated ready-to-cook Asian moon scallop (RAM) during refrigerated storage at 4 °C for 3 days under different antimicrobial treatments. Treatments included inoculated RAM without antimicrobial treatment (I + RAM), inoculated RAM treated with chitooligosaccharide–epigallocatechin gallate conjugate (CEGC; final concentration of 313 μg/mL) (I + RAM + CEGC), inoculated RAM treated with nisin (final concentration of 2500 μg/mL) (I + RAM + Nisin), and inoculated RAM treated with the combination of CEGC (final concentration of 78 μg/mL) and nisin (final concentration of 625 μg/mL), corresponding to a fractional inhibitory concentration index (FICI) of 0.50 (I + RAM + CEGC + Nisin). Bacterial populations are expressed as log CFU/g. Values are presented as mean ± standard deviation (SD) from triplicate experiments (*n* = 3). The detection limit was <2 log CFU/g, and N.D. indicates non-detected cells. Different uppercase letters on the bars indicate significant differences during storage within the same treatment, whereas different lowercase letters on the bars indicate significant differences among treatments at the same storage time (*p* < 0.05).

As shown in Figure 7, the population of *V. parahaemolyticus* in the untreated inoculated control remained relatively stable throughout storage, decreasing only slightly from 6.73 to 6.57 log CFU/g, indicating that cold storage alone was insufficient to effectively control the pathogen. Treatment with CEGC alone (313 µg/mL) significantly reduced the bacterial population from 6.03 to 3.09 log CFU/g after 3 days of storage, corresponding to approximately 2.94 log CFU/g reduction. Similarly, nisin treatment alone (2500 µg/mL) reduced the bacterial counts from 5.80 to 3.07 log CFU/g, representing a reduction of approximately 2.73 log CFU/g. Notably, the combined treatment of CEGC and nisin at synergistic concentrations (78 µg/mL CEGC + 625 µg/mL nisin) exhibited the strongest antibacterial effect, reducing *V. parahaemolyticus* to undetectable levels (<2 log CFU/g) after 3 days of refrigerated storage. This inhibitory effect of combined treatments was significantly greater than those observed for that obtained by the use of individual antimicrobial agent. More importantly, substantially lower concentrations of both antimicrobial agents were used in the combined treatments. The present results demonstrated that the combination of CEGC and nisin effectively controlled *V. parahaemolyticus* in ready-to-cook Asian moon scallop under refrigerated conditions. Although refrigeration at 4 °C slightly suppressed bacterial growth, the pathogen remained highly viable in untreated samples, highlighting the psychrotrophic tolerance and persistence of *V. parahaemolyticus* in seafood products during refrigerated storage. The enhanced activity might result from complementary mechanisms of action between the two compounds. Another important observation was that the synergistic treatment remained highly effective within the complex seafood matrix. Food components such as proteins, lipids, and minerals often reduce antimicrobial efficacy by interacting with active compounds [56]. Nevertheless, the CEGC–nisin combination retained strong antibacterial activity under refrigerated conditions, suggesting potential applicability in real food systems. The ability to achieve complete inhibition at substantially reduced concentrations was advantageous for minimizing potential impacts on sensory quality and reducing the costs in seafood preservation. The combination of nisin and phytic acid significantly enhanced antimicrobial activity against pathogenic *Escherichia coli* O157:H7 in food systems [50]. Therefore, the synergistic action between CEGC and nisin observed in the present study represented a promising natural preservation strategy for improving the microbial safety and shelf life extension of ready-to-cook seafood products, particularly Asian moon scallop.

## 4. Conclusions

Ready-to-cook Asian moon scallop (RAM) was demonstrated to serve as a reservoir of seafood-associated spoilage and opportunistic pathogenic bacteria, including *Vibrio*, *Shewanella*, and *Aeromonas* spp., with considerable proteolytic, hemolytic, and biofilm-forming capacities. The antimicrobial activity of CEGC was stronger than that of nisin, with a MIC of 313 μg/mL, whereas the MIC of nisin ranged from 2500 to 5000 μg/mL. The combination of CEGC and nisin enhanced antibacterial activity, with synergistic effects observed against *V. parahaemolyticus* and *V. fluvialis* (FICI = 0.50). The combined treatment also reduced the required concentrations of both antimicrobial agents and effectively disrupted bacterial membrane integrity and inhibited biofilm formation. In inoculated RAM, the CEGC–nisin combination reduced *V. parahaemolyticus* populations during refrigerated storage at 4 °C, demonstrating its potential as a natural antimicrobial treatment for minimally processed seafood. Further studies are needed to evaluate the long-term antimicrobial efficacy, sensory quality, and stability of the CEGC–nisin treatment under different processing and storage conditions. Future research should also elucidate the molecular mechanisms underlying the synergistic interaction between CEGC and nisin and validate the effectiveness, consistency, and feasibility of the combined treatment at pilot-scale and commercial processing conditions.

## Figures and Tables

**Table 1:** Minimum inhibitory concentrations (MICs), combined MICs, fractional inhibitory concentration indices (FICIs), and interaction profiles of chitooligosaccharide–epigallocatechin gallate conjugate (CEGC) and nisin against seafood-associated pathogenic and spoilage bacteria.

Bacterial Strains	Chitooligosaccharide– Epigallocatechin Gallate Conjugate (CEGC)		Nisin		FICI	Interpretation
	Single MIC (µg/mL)	Combined MIC (µg/mL)	Single MIC (µg/mL)	Combined MIC (µg/mL)		
*Vibrio parahaemolyticus* (VP)	313	78	2500	625	0.50	Synergism
*Vibrio fluvialis* (VF)	313	78	5000	1250	0.50	Synergism
*Vibrio alginolyticus* (VA)	313	156	2500	1250	1.00	Additive
*Aeromonas hydrophila* (AH)	313	313	5000	313	1.06	Indifferent
*Aeromonas veronii* (AV)	313	156	2500	1250	1.00	Additive
*Shewanella putrefaciens* (SP)	313	156	2500	1250	1.00	Additive

## Data Availability

The original contributions presented in the study are included in the article; further inquiries can be directed to the corresponding author.

## Supplementary Materials

The following supporting information can be downloaded at: https://www.mdpi.com/article/10.3390/foods15173072/s1, Table S1: Identification and antimicrobial susceptibility profiles of 102 seafood-associated bacterial isolates obtained using selective culture media. Presumptive isolates of *Vibrio* spp., *Shewanella* spp., and *Aeromonas* spp. were identified by matrix-assisted laser desorption/ionization time-of-flight mass spectrometry (MALDI-TOF MS).

## Abbreviations

APC	Aerobic plate count
BAM	Bacteriological Analytical Manual
CEGC	Chitooligosaccharide–epigallocatechin gallate conjugate
CFU	Colony-forming unit
CLSM	Confocal laser scanning microscopy
COS	Chitooligosaccharide
CV	Crystal violet
EGCG	Epigallocatechin gallate
EPS	Extracellular polymeric substance
FICI	Fractional inhibitory concentration index
MALDI-TOF/MS	Matrix-assisted laser desorption/ionization time-of-flight mass spectrometry
MBC	Minimum bactericidal concentration
MHB	Mueller–Hinton broth
MIC	Minimum inhibitory concentration
PCA	Principal component analysis
PI	Propidium iodide
RAM	Ready-to-cook Asian moon scallop
SEM	Scanning electron microscopy
TSA	Tryptic soy agar
TSB	Tryptic soy broth
TCBS	Thiosulfate–citrate–bile salts–sucrose agar
