# Supplementary material for: Synergistic Effects Between Chitooligosaccharide–Epigallocatechin Gallate Conjugate and Nisin on Inhibition of Seafood-Associated Vibrio spp., Aeromonas spp., and Shewanella spp
Source: Foods. 2026 Aug 30;15(17):3072. doi: 10.3390/foods15173072 (PMC13565219; doi:10.3390/foods15173072)
Supplement: Supplementary file 1 [file foods-15-03072-s001.zip › foods-4515294-supplementary.pdf]

Supplementary Table S1. Identification and antimicrobial susceptibility profiles of 102 seafood-associated bacterial isolates obtained using selective culture media. Presumptive isolates of *Vibrio* spp., *Shewanella* spp., and *Aeromonas* spp. were identified by matrix-assisted laser desorption/ionization time-of-flight mass spectrometry (MALDI-TOF MS).

Isolate No.	Selective media for isolation	MALDI-TOF MS identification/ [Identification score]	CEGC (MIC, µg/mL)	CEGC (MBC, µg/mL)	Nisin (MIC, µg/mL)	Nisin (MBC, µg/mL)
1	*Vibrio* spp. presumptive	*Vibrio parahaemolyticus* [2.34]	313	1250	2500	5000
2	*Vibrio* spp. presumptive	*Shewanella indica* [2.10]	625	625	2500	5000
3	*Vibrio* spp. presumptive	*Shewanella algae* [2.19]	313	1250	2500	2500
4	*Vibrio* spp. presumptive	*Vibrio parahaemolyticus* [2.10]	156	625	2500	2500
5	*Vibrio* spp. presumptive	*Vibrio parahaemolyticus* [2.27]	313	1250	2500	5000
6	*Vibrio* spp. presumptive	*Vibrio parahaemolyticus* [2.20]	156	625	2500	2500
7	*Vibrio* spp. presumptive	*Vibrio parahaemolyticus* [2.30]	156	625	2500	2500
8	*Vibrio* spp. presumptive	*Vibrio parahaemolyticus* [2.10]	313	1250	2500	5000
9	*Vibrio* spp. presumptive	*Vibrio parahaemolyticus* [2.10]	156	625	2500	2500
10	*Vibrio* spp. presumptive	*Vibrio parahaemolyticus* [2.10]	313	1250	2500	5000
11	*Vibrio* spp. presumptive	*Vibrio parahaemolyticus* [2.27]	313	1250	2500	5000
12	*Vibrio* spp. presumptive	*Vibrio parahaemolyticus* [2.10]	313	1250	2500	5000
13	*Vibrio* spp. presumptive	*Vibrio harveyi* [2.32]	313	1250	2500	5000
14	*Vibrio* spp. presumptive	*Vibrio harveyi* [2.30]	313	1250	2500	5000
15	*Vibrio* spp. presumptive	*Vibrio parahaemolyticus* [2.24]	313	1250	2500	5000
16	*Vibrio* spp. presumptive	*Vibrio parahaemolyticus* [2.05]	156	625	2500	2500
17	*Vibrio* spp. presumptive	*Vibrio harveyi* [2.20]	313	1250	2500	5000
18	*Vibrio* spp. presumptive	*Vibrio parahaemolyticus* [2.13]	313	1250	2500	5000
19	*Vibrio* spp. presumptive	*Vibrio parahaemolyticus* [2.44]	313	1250	2500	5000
20	*Vibrio* spp. presumptive	*Vibrio harveyi* [2.13]	313	1250	2500	5000
21	*Vibrio* spp. presumptive	*Vibrio parahaemolyticus* [2.07]	156	625	2500	2500
22	*Vibrio* spp. presumptive	*Vibrio harveyi* [2.05]	313	313	2500	2500
23	*Vibrio* spp. presumptive	*Vibrio parahaemolyticus* [2.32]	156	625	2500	2500
24	*Vibrio* spp. presumptive	*Vibrio harveyi* [2.16]	313	625	2500	2500
25	*Vibrio* spp. presumptive	*Vibrio parahaemolyticus* [2.30]	156	625	2500	2500
26	*Vibrio* spp. presumptive	*Vibrio harveyi* [2.30]	313	313	2500	5000
27	*Vibrio* spp. presumptive	*Vibrio parahaemolyticus* [2.12]	156	625	2500	2500
28	*Vibrio* spp. presumptive	*Vibrio parahaemolyticus* [2.08]	313	1250	2500	5000
29	*Vibrio* spp. presumptive	*Vibrio parahaemolyticus* [2.10]	156	625	2500	2500
30	*Vibrio* spp. presumptive	*Vibrio parahaemolyticus* [2.15]	313	1250	2500	5000
31	*Vibrio* spp. presumptive	*Vibrio harveyi* [2.24]	625	1250	2500	5000
32	*Vibrio* spp. presumptive	*Vibrio harveyi* [2.20]	313	1250	2500	2500
33	*Vibrio* spp. presumptive	*Vibrio anguillarum* [2.11]	625	1250	2500	5000
34	*Vibrio* spp. presumptive	*Exiguobacterium* sp. [1.85]	625	1250	2500	5000
35	*Vibrio* spp. presumptive	*Vibrio alginolyticus* [2.10]	313	1250	2500	5000
36	*Vibrio* spp. presumptive	*Vibrio alginolyticus* [2.34]	625	1250	2500	5000
37	*Vibrio* spp. presumptive	*Vibrio alginolyticus* [2.27]	313	1250	2500	5000
38	*Vibrio* spp. presumptive	*Vibrio alginolyticus* [2.10]	313	1250	2500	5000
39	*Vibrio* spp. presumptive	*Vibrio alginolyticus* [2.24]	313	1250	2500	5000
40	*Vibrio* spp. presumptive	*Vibrio alginolyticus* [2.10]	625	1250	2500	2500
41	*Vibrio* spp. presumptive	*Vibrio furnissii* [2.32]	625	1250	2500	2500
42	*Vibrio* spp. presumptive	*Proteus hauseri* [2.10]	625	1250	2500	2500
43	*Vibrio* spp. presumptive	*Aeromonas veronii* [2.17]	625	1250	2500	5000
44	*Vibrio* spp. presumptive	*Vibrio alginolyticus* [2.44]	313	1250	2500	2500

45	*Vibrio* spp. presumptive	*Vibrio alginolyticus* [2.16]	313	1250	2500	2500
46	*Vibrio* spp. presumptive	*Vibrio alginolyticus* [2.32]	156	625	2500	2500
47	*Vibrio* spp. presumptive	*Vibrio parahaemolyticus* [2.08]	156	625	2500	2500
48	*Vibrio* spp. presumptive	*Vibrio alginolyticus* [2.20]	313	625	2500	2500
49	*Vibrio* spp. presumptive	*Unidentified* [1.57]	-	-	-	-
50	*Vibrio* spp. presumptive	*Vibrio alginolyticus* [2.15]	313	1250	2500	5000
51	*Vibrio* spp. presumptive	*Vibrio parahaemolyticus* [2.17]	156	625	2500	2500
52	*Vibrio* spp. presumptive	*Vibrio harveyi* [2.11]	625	1250	2500	2500
53	*Vibrio* spp. presumptive	*Vibrio navarrensis* [2.16]	313	1250	2500	2500
54	*Vibrio* spp. presumptive	*Vibrio europaeus* [2.20]	313	1250	2500	5000
55	*Vibrio* spp. presumptive	*Vibrio europaeus* [2.30]	313	1250	2500	5000
56	*Vibrio* spp. presumptive	*Vibrio europaeus* [2.20]	625	1250	2500	5000
57	*Vibrio* spp. presumptive	*Vibrio parahaemolyticus* [2.19]	156	625	2500	2500
58	*Vibrio* spp. presumptive	*Vibrio alginolyticus* [2.34]	313	1250	2500	5000
59	*Vibrio* spp. presumptive	*Vibrio fluvialis* [2.11]	625	1250	5000	5000
60	*Vibrio* spp. presumptive	*Vibrio natriegens* [2.11]	625	1250	2500	2500
61	*Vibrio* spp. presumptive	*Vibrio parahaemolyticus* [2.27]	156	625	2500	2500
62	*Vibrio* spp. presumptive	*Vibrio parahaemolyticus* [2.13]	313	1250	2500	2500
63	*Vibrio* spp. presumptive	*Vibrio parahaemolyticus* [2.32]	156	625	2500	2500
64	*Vibrio* spp. presumptive	*Aeromonas eucrenophila* [2.20]	313	313	2500	5000
65	*Shewanella* spp. presumptive	*Shewanella* sp. [1.91]	625	625	2500	5000
66	*Shewanella* spp. presumptive	*Shewanella indica* [2.13]	625	625	2500	5000
67	*Shewanella* spp. presumptive	*Shewanella algae* [2.13]	313	313	2500	2500
68	*Shewanella* spp. presumptive	*Shewanella algae* [2.30]	313	313	2500	5000
69	*Shewanella* spp. presumptive	*Shewanella indica* [2.32]	625	625	2500	5000
70	*Shewanella* spp. presumptive	*Shewanella indica* [2.10]	625	625	2500	5000
71	*Shewanella* spp. presumptive	*Shewanella indica* [2.10]	313	313	2500	5000
72	*Shewanella* spp. presumptive	*Shewanella* sp. [1.86]	625	625	2500	5000
73	*Shewanella* spp. presumptive	*Shewanella algae* [2.11]	313	313	2500	2500
74	*Shewanella* spp. presumptive	*Shewanella indica* [2.05]	313	313	2500	2500
75	*Shewanella* spp. presumptive	*Shewanella* sp. [1.85]	625	625	2500	5000
76	*Shewanella* spp. presumptive	*Shewanella* sp. [1.90]	625	625	2500	5000
77	*Shewanella* spp. presumptive	*Unidentified* [1.62]	-	-	-	-
78	*Shewanella* spp. presumptive	*Unidentified* [1.68]	-	-	-	-
79	*Shewanella* spp. presumptive	*Unidentified* [1.55]	-	-	-	-
80	*Shewanella* spp. presumptive	*Shewanella* sp. [1.84]	313	625	2500	5000
81	*Shewanella* spp. presumptive	*Unidentified* [1.53]	-	-	-	-
82	*Shewanella* spp. presumptive	*Unidentified* [1.49]	-	-	-	-

83	*Aeromonas* spp. presumptive	*Aeromonas eucrenophila* [2.30]	313	313	2500	5000
84	*Aeromonas* spp. presumptive	*Aeromonas bestiarum* [2.30]	625	625	5000	5000
85	*Aeromonas* spp. presumptive	*Aeromonas bestiarum* [2.32]	313	313	5000	5000
86	*Aeromonas* spp. presumptive	*Aeromonas eucrenophila* [2.20]	313	625	5000	5000
87	*Aeromonas* spp. presumptive	*Aeromonas salmonicida* [2.20]	313	313	2500	5000
88	*Aeromonas* spp. presumptive	*Aeromonas veronii* [2.13]	625	625	2500	5000
89	*Aeromonas* spp. presumptive	*Aeromonas hydrophila* [2.15]	313	313	5000	5000
90	*Aeromonas* spp. presumptive	*Aeromonas bestiarum* [2.08]	313	625	2500	5000
91	*Aeromonas* spp. presumptive	*Aeromonas bestiarum* [2.05]	313	313	2500	5000
92	*Aeromonas* spp. presumptive	*Aeromonas veronii* [2.24]	625	625	2500	5000
93	*Aeromonas* spp. presumptive	*Aeromonas eucrenophila* [2.23]	625	625	2500	5000
94	*Aeromonas* spp. presumptive	*Aeromonas eucrenophila* [2.25]	625	625	2500	5000
95	*Aeromonas* spp. presumptive	*Aeromonas eucrenophila* [2.11]	625	625	2500	5000
96	*Aeromonas* spp. presumptive	*Aeromonas hydrophila* [2.11]	625	625	5000	5000
97	*Aeromonas* spp. presumptive	*Aeromonas bestiarum* [2.05]	625	625	5000	5000
98	*Aeromonas* spp. presumptive	*Aeromonas eucrenophila* [2.27]	625	625	5000	5000
99	*Aeromonas* spp. presumptive	*Aeromonas bestiarum* [2.15]	313	313	5000	5000
100	*Aeromonas* spp. presumptive	*Aeromonas eucrenophila* [2.27]	313	313	5000	5000
101	*Aeromonas* spp. presumptive	*Aeromonas bestiarum* [2.20]	313	313	5000	5000
102	*Aeromonas* spp. presumptive	*Aeromonas bestiarum* [2.10]	313	313	2500	5000
VP	Standard strain	*Vibrio parahaemolyticus* ATCC 17802	313	1250	2500	5000
VF	Standard strain	*Vibrio fluvialis* BL186	313	625	5000	5000
VA	Standard strain	*Vibrio alginolyticus* DMST 14800	313	1250	2500	2500
AH	Standard strain	*Aeromonas hydrophila* ATCC 7966	313	313	5000	5000
AV	Standard strain	*Aeromonas veronii* ATCC 35624	313	625	2500	5000
SP	Standard strain	*Shewanella putrefaciens JCM 20190*	313	313	2500	2500

Antimicrobial susceptibility was evaluated by determining the minimum inhibitory concentration (MIC) and minimum bactericidal concentration (MBC) of chitooligosaccharide–epigallocatechin gallate conjugate (CEGC) and nisin against each isolate. Reference strains included *Vibrio parahaemolyticus* ATCC 17802 (VP), *Vibrio fluvialis* BL186 (VF), *Vibrio alginolyticus* DMST 14800 (VA), *Aeromonas hydrophila* ATCC 7966 (AH), *Aeromonas veronii* ATCC 35624 (AV), and *Shewanella putrefaciens* JCM 20190 (SP). Antimicrobial concentrations are expressed as µg/mL.

“Unidentified” indicates isolates that could not be reliably assigned to a bacterial species by MALDI-TOF MS analysis.
